# Spatial single-cell profiling and neighbourhood analysis reveal the determinants of immune architecture connected to checkpoint inhibitor therapy outcome in hepatocellular carcinoma

**DOI:** 10.1136/gutjnl-2024-332837

**Published:** 2024-09-30

**Authors:** Henrike Salié, Lara Wischer, Antonio D’Alessio, Ira Godbole, Yuan Suo, Patricia Otto-Mora, Juergen Beck, Olaf Neumann, Albrecht Stenzinger, Peter Schirmacher, Claudia A M Fulgenzi, Andreas Blaumeiser, Melanie Boerries, Natascha Roehlen, Michael Schultheiß, Maike Hofmann, Robert Thimme, David J Pinato, Thomas Longerich, Bertram Bengsch

**Affiliations:** 1 Department of Internal Medicine II, Medical Center - University of Freiburg, Freiburg, Germany; 2 Department of Surgery & Cancer, Imperial College London, London, UK; 3 Department of Translational Medicine, University of Piemonte Orientale, Novara, Italy; 4 Institute of Pathology, University Hospital Heidelberg, Heidelberg, Germany; 5 Institute of Medical Bioinformatics and Systems Medicine, University of Freiburg, Freiburg im Breisgau, Germany; 6 German Cancer Consortium (DKTK), Heidelberg, Germany, partner site Freiburg, Freiburg, Germany; 7 Signalling Research Centres BIOSS and CIBSS, Freiburg, Germany

**Keywords:** HEPATOCELLULAR CARCINOMA, IMMUNOTHERAPY, LIVER IMMUNOLOGY, CANCER IMMUNOBIOLOGY, IMAGE ANALYSIS

## Abstract

**Background:**

The determinants of the response to checkpoint immunotherapy in hepatocellular carcinoma (HCC) remain poorly understood. The organisation of the immune response in the tumour microenvironment (TME) is expected to govern immunotherapy outcomes but spatial immunotypes remain poorly defined.

**Objective:**

We hypothesised that the deconvolution of spatial immune network architectures could identify clinically relevant immunotypes in HCC.

**Design:**

We conducted highly multiplexed imaging mass cytometry on HCC tissues from 101 patients. We performed in-depth spatial single-cell analysis in a discovery and validation cohort to deconvolute the determinants of the heterogeneity of HCC immune architecture and develop a spatial immune classification that was tested for the prediction of immune checkpoint inhibitor (ICI) therapy.

**Results:**

Bioinformatic analysis identified 23 major immune, stroma, parenchymal and tumour cell types in the HCC TME. Unsupervised neighbourhood detection based on the spatial interaction of immune cells identified three immune architectures with differing involvement of immune cells and immune checkpoints dominated by either CD8 T-cells, myeloid immune cells or B- and CD4 T-cells. We used these to define three major spatial HCC immunotypes that reflect a higher level of intratumour immune cell organisation: depleted, compartmentalised and enriched. Progression-free survival under ICI therapy differed significantly between the spatial immune types with improved survival of enriched patients. In patients with intratumour heterogeneity, the presence of one enriched area governed long-term survival.

**WHAT IS ALREADY KNOWN ON THIS TOPIC**

- Hepatocellular carcinoma (HCC) is a heterogeneous tumour entity with a response of immunotherapy observed only in a fraction of patients. Distinct immune populations have been connected to immunotherapy outcomes but lack clinical translation.

**WHAT THIS STUDY ADDS**

- Our spatially resolved single-cell analysis of the heterogeneous HCC microenvironment identifies the determinants of HCC immune architecture. With this information, we derive a spatial immune classification suitable for clinical translation that correlates with immune checkpoint inhibitor therapy outcomes. Moreover, our work points to different immunoregulatory mechanisms active in distinct immunotypes.

**HOW THIS STUDY MIGHT AFFECT RESEARCH, PRACTICE OR POLICY**

- The spatial immune classification may facilitate the prediction of immunotherapy outcomes in HCC patients, influence rationale choice of therapy as well as combinatorial regimens and may guide immunotype-directed therapy development.

## Introduction

 The impact of cellular interactions in the tumour microenvironment (TME) on immunotherapy outcomes remains a major question in many cancers, in particular in those with high tumour heterogeneity, such as hepatocellular carcinoma (HCC). The spatial organisation of the antitumour immune response is emerging as an important component of the cancer-immune cycle that governs the success of immunotherapy.^1^ Nevertheless, spatial immunotypes currently remain poorly defined in several cancer entities.

HCC is the most common primary liver cancer that ranks third in cancer-related mortality globally.^2^ HCC patients are frequently diagnosed at an advanced stage with indication for systemic therapy.^3 4^ The current immune checkpoint inhibitor (ICI) based first-line treatment targets programmed death ligand (PD-L1) alone or in combination with vascular endothelial growth factor (VEGF)^5 6^ or cytotoxic T-lymphocyte antigen 4 (CTLA-4).^7^ Despite significantly better therapy outcomes compared with tyrosine kinase inhibitor (TKI) therapy, objective response rates (ORR) to ICI range from <20% for ICI monotherapy^7 8^ to 30% for combination therapy.^6^ Thus, a large proportion of patients do not respond to current ICI therapies. The mechanisms underlying therapeutic efficacy remain poorly understood and currently no validated biomarkers exist to predict the response of HCC patients to ICI therapy.^9 10^ Nevertheless, several studies point to a role of immunological features identified in the HCC TME to correlate with ICI therapy outcomes, such as elevated presence of immune signatures, infiltration with CD8 T-cells and a contribution of CD4+ regulatory T-cells.^11^,^14^ Our previous work identified the differentiation of CD8 T-cells in the HCC TME into exhausted (TEX) or tissue-resident memory (TRM) cells as a determinant of therapy outcomes.^12^ Additional evidence from murine models of metabolic disease-associated HCC suggests that the quality of the T-cell response may be correlated with disease aetiology and impact the outcome of checkpoint therapy.^15 16^ Collectively, these data indicate that the immune architecture of HCC is likely connected to therapeutic outcomes.

However, previous approaches to classify HCCs based on immune features obtained from transcriptomic or proteomic tissue analysis, such as molecular classifications,^17^,^22^ did not directly predict outcomes to checkpoint therapy. This is possibly related to the lack of single-cell granularity and spatial context in these studies and overlapping expression programmes in distinct tumour and immune cells. Novel methodological approaches, such as highly multiplexed imaging mass cytometry (IMC), now allow a precise characterisation of tumour and immune cell types using single-cell proteomics while retaining spatial information to deconvolute tumour-immune ecosystems.^23^

We postulated that the composition and the spatial organisation of the immune infiltrate at a single-cell level within the HCC TME may provide insights into immune escape mechanisms and predict response to ICI therapy. We speculated that a precise understanding of the HCC immune architecture can be used to determine outcome-associated immunotypes.

Thus, we set out to characterise the immune architecture of the HCC TME using spatially resolved, high-dimensional single-cell-based immune network analysis. Our work identified distinct immune cell types involved in microenvironmental interactions that determine three major immunotypes of HCC in a discovery cohort. We validated the immunotype classification in an independent cohort and identified an immunotype that correlates with immunotherapy outcome.

## Results

### Deep single-cell spatial profiling of the HCC TME

**Figure 1:**
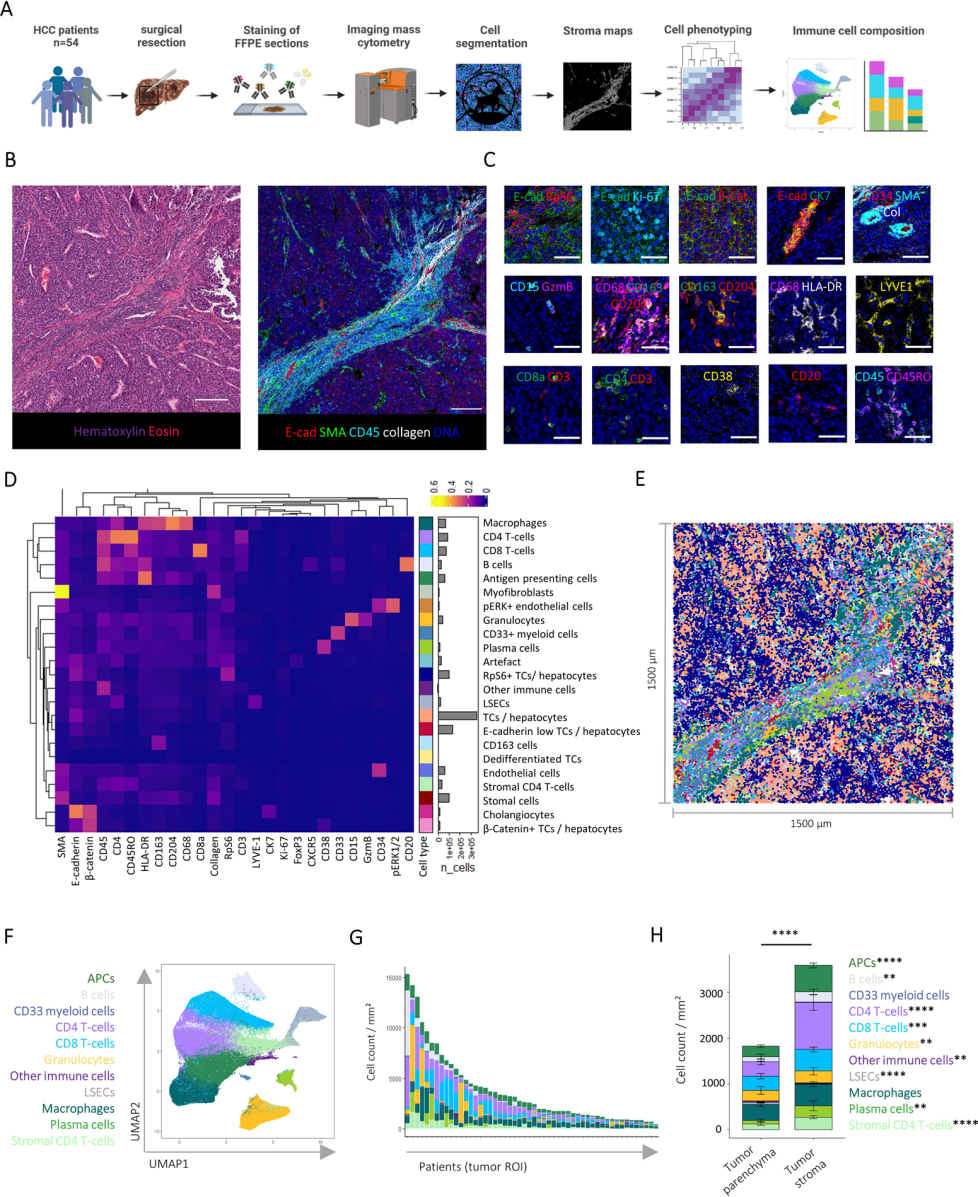
Imaging mass cytometry of the hepatocellular carcinoma tumour microenvironment. (**A**) Workflow illustrating IMC data preparation and analysis steps. (**B**) Example H&E (left) and respective IMC composite image (right) from one HCC patient. Scale bars indicate 200 µm (**C**) Example composite images visualising key cellular components of the HCC TME. Scale bars indicate 50 µm. (**D**) Heatmap visualising mean marker expression of detected cell types after channel normalisation and PhenoGraph clustering. (**E**) Cell types on example HCC image. (**F**) UMAP representation coloured by detected immune cell types. (**G**) Amount and distribution of immune cell types in the tumour ROI from each patient. (**H**) Stacked bar plots visualising mean immune cell type densities in the tumour parenchyma compared with intratumour stroma. Error bars indicate SE. Significant differences were determined by paired Wilcoxon tests and Bonferroni corrected for multiple comparisons. ns, not significant, **p<0.01, ***p<0.001, ****p<0.0001. FFPE, formalin-fixed paraffine-embedded; HCC, hepatocellular carcinoma; IMC, imaging mass cytometry; ROI, regions of interest; TME, tumour microenvironment.

To deconvolute the spatial organisation of the HCC TME, we developed a 41-plex IMC panel suitable for identifying key immune cell populations, immune checkpoints, hepatic parenchymal cells and tumour markers (online supplemental table 1). First, we analysed tumour tissue from 54 HCC patients obtained during surgical resection (figure 1A). Spatial expression data were obtained after laser ablation and mass spectrometry analysis of metal isotopes resulting in highly multiplexed images with a resolution of 1 μm^2^ (figure 1B). Image inspection displayed expected marker expression profiles for parenchymal, structural and immune cells in our cohort (figure 1C). We extracted single-cell level data from our images by artificial intelligence (AI)-aided cell segmentation followed by channel normalisation. PhenoGraph clustering analysis resulted in the detection of 23 cell types in the HCC and liver microenvironment including tumour and hepatocyte cell types (E-cadherin+, β-Catenin+, RpS6+, dedifferentiated), stromal cell types (endothelial cells (CD34+), myofibroblasts (a-SMA+), stromal cells (collagen+)), as well as major immune cell types, including adaptive (eg, CD8 (CD3+CD8+), CD4 T-cells (CD3+CD4+), B cells (CD20+), plasma cells (CD38+)) and innate (eg, macrophages (CD68+), antigen-presenting cells (APCs) (HLA-DR+), granulocytes (CD15+) and CD33+myeloid cells (CD33+, CD68+)) (figure 1D) and their spatial organisation (figure 1E). In sum, our approach identified a diverse range of tumour, stroma and immune cell types at the spatial single-cell level in the HCC TME.

### Significant heterogeneity in the quantity and quality of TMEs in HCC

To understand the immune architecture of the HCC TME, we focused on the identified immune cell populations. As shown in figure 1F,G, comparison of the amount and composition of immune cells in the tumour regions of interest (ROIs) between patients revealed profound quantitative and qualitative heterogeneity between HCC specimens. To independently analyse distinct microenvironmental compartments within the TME, we created masks mapping each cell’s location within the intratumoural stroma or tumour parenchyma (online supplemental figure 1A). Of note, the cellular composition differed significantly between the tumour parenchyma and the stroma (figure 1H). Overall immune cell density was higher in the tumour stroma, particularly driven by an increase in CD4 and CD8 T-cells, APCs and B cells (figure 1H). This analysis identified a significant immune heterogeneity between HCCs. It also highlighted the presence of intratumour microenvironmental niches possibly influencing a differential immune cell infiltration, such as the tumour stroma.

### Deconvolution of the immune architecture reveals three major immune neighbourhoods

**Figure 2:**
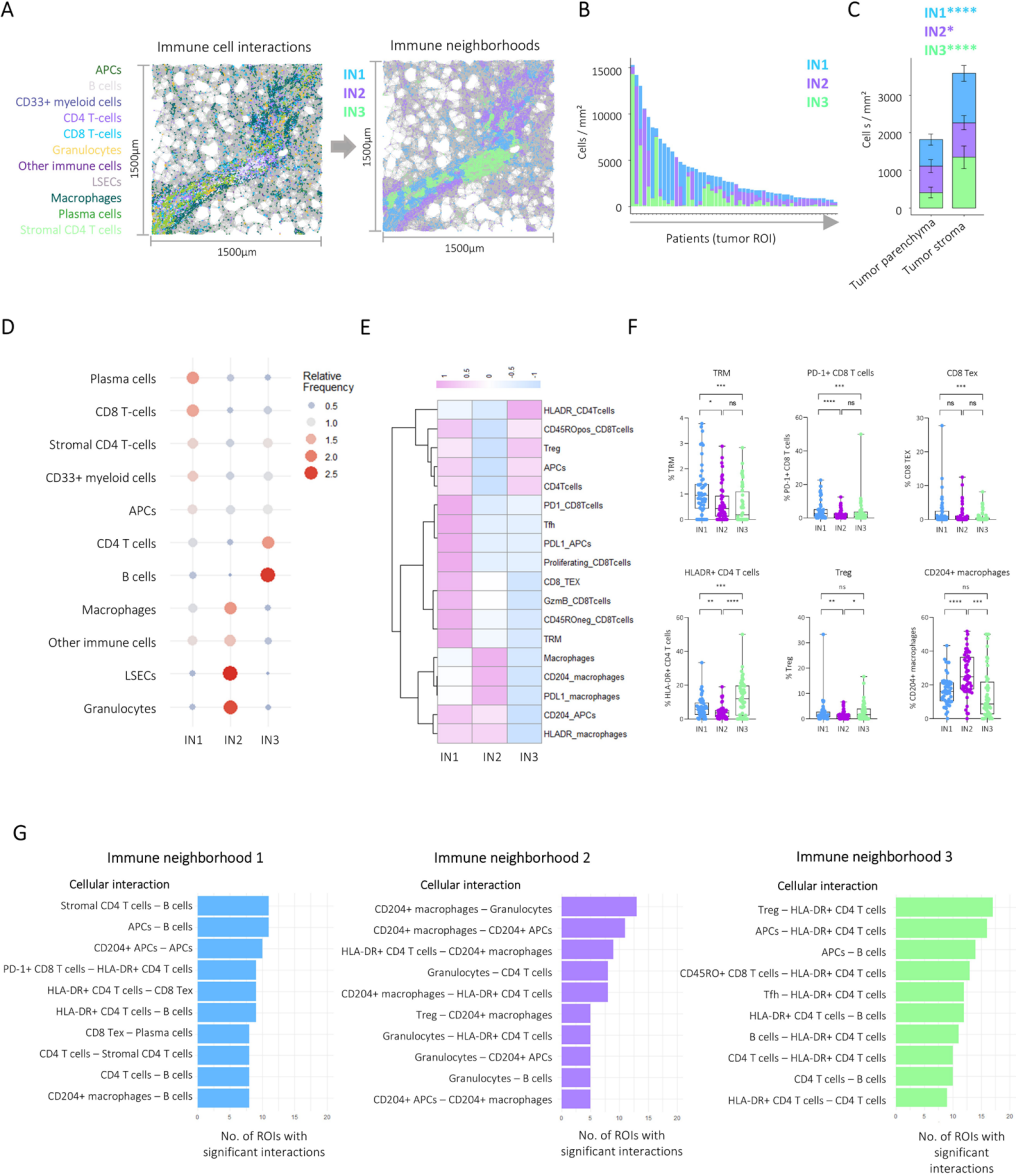
Spatial profiling of the hepatocellular carcinoma tumour immune microenvironment reveals distinct immune networks. (**A**) Immune cell type interactions (left image) were used to cluster immune cells into three immune neighbourhoods (right image). (**B**) Stacked bar plots visualising mean immune neighbourhood composition of each patient. (**C**) Stacked bar plots visualising mean immune neighbourhood composition per intratumour compartment. Error bars indicate SE of the mean. Wilcoxon tests were used to assess statistical significance. (**D**) Bubble heatmap illustrating the relative frequency of immune cell types in detected immune neighbourhoods. (**E**) Heatmap visualising z-scored frequency of gated immune subsets. (**F**) Boxplots comparing gated immune subset frequencies between immune neighbourhoods. Pairwise comparisons were performed using Wilcoxon tests and Bonferroni corrected for multiple comparisons. (**G**) Pairwise immune subset interactions were calculated for each immune neighbourhood separately. Bar plots show the 10 most frequent significant immune cell interactions. *p<0.05, **p<0.01, ***p<0.001, ****p<0.0001. APCs, antigen-presenting cells; CD8 Tex, exhausted CD8 T cells; ns, not significant; ROI, region of interest; Treg, regulatory T cells; TRM, tissue-resident memory T cells.

To dissect the HCC immune architecture in a data-driven manner, we first performed k-means clustering of immune cells based on their spatial proximity. This approach revealed three distinct immune neighbourhoods (INs) (figure 2A) that varied between patients (figure 2B) and intratumour microanatomic niches (figure 2C). Analysis of the cellular contributions to these neighbourhoods revealed that IN1 was enriched for CD8 T-cells and plasma cells, IN2 was enriched for myeloid cells with prominent infiltration of macrophages and granulocytes and IN3 was enriched for B cells and CD4 T-cells (figure 2D, online supplemental figure 1).

To understand whether the INs were connected with the presence of specific immune cell subtypes potentially linked to distinct tumour immune escape mechanisms, we analysed the contribution of T-cell and myeloid subsets, including PD-1+CD8 T-cells, exhausted CD8 T-cells expressing PD-1 together with additional immune checkpoints (eg, Tim-3, Lag-3) (CD8 TEX), tissue-resident memory T-cells (TRM), regulatory T-cells (Treg), CD204+and PD-L1+M2 like macrophages and APCs (online supplemental figure 1). This analysis revealed an enrichment of TRM, PD-1+CD8 T-cells, CD8 TEX and CD45RO− CD8 T-cells in IN1, whereas CD204+M2 like macrophages were significantly increased in IN2. In contrast, IN3 showed the highest frequency of HLA-DR+CD4 T-cells. Tregs were predominantly identified in IN1 and IN3 neighbourhoods (figure 2E,F). These results demonstrate that the identified INs were linked to the infiltration of distinct immune cell subtypes.

We hypothesised that relevant immunological cell interactions were characterised by distinct interaction patterns on the spatial single-cell level. Permutation test-based interaction analyses within each neighbourhood were performed on a single-cell level and analysed by immune cell subset (figure 2G). This analysis revealed clear interaction patterns in the identified INs. For example, in IN1, the most frequent significant interactions were observed between different activated adaptive immune subsets and APCs, such as HLA-DR+CD4 T-cell interactions with PD-1+CD8 T-cells and severely exhausted PD-1+CD8 T-cells that express further inhibitory receptors (CD8 TEX), B cell interactions with APCs and CD8 TEX cell interactions with plasma cells, suggesting an active antitumour immune response. In contrast, IN2 showed predominant interactions of M2-like myeloid subsets with each other and CD4 T-cells with a prominent role of CD204+macrophages and CD204+APCs, hinting towards an immunosuppressive environment. IN3 was dominated by interactions between subsets of antigen-presenting cells like B cells, HLA-DR+CD4 T-cells, T follicular helper (Tfh) cells and APCs with a reduced role of cytotoxic CD8 T-cells in comparison to IN1. Interestingly, Tregs interacting with HLA-DR+CD4 T-cells represented the most frequent interaction in IN3, in contrast to IN1, suggesting that the interactions in IN3 could also be inhibitory.

Innate lymphoid cells (ILC) and innate-like T cells may also contribute significantly to HCC antitumour immunity.^24^ Mucosal associated invariant T (MAIT) cells and natural killer (NK) cells were assessed using an IMC staining approach compatible with fresh-frozen HCC tissue sections that preserve key MAIT and NK cell markers (online supplemental figure 2A, B). NK cells (CD7+CD56+CD3−) and MAIT cells (CD3+CD161+IL18-R+) were identified as separate immune clusters and were significantly enriched in the tumour stroma compared with the parenchyma (online supplemental figure 2C–F). NK cell density correlated with MAIT cell and plasma cell density as well as with myeloid cell subsets (neutrophils, Kupffer cells, CD56+monocytes) and B cell density (online supplemental figure 2G). NK cells interacted mainly with myeloid cell subsets (neutrophils, macrophages and Kupffer cells) as well as CD4 and CD8 T cells while avoiding B cells and APCs (online supplemental figure 2H). MAIT cell density correlated with B cells and plasma cells as well as NK cells and other lymphocytes (online supplemental figure 2G). They interacted mainly with CD4 and CD8 T cells (online supplemental figure 2H). Collectively, these data point to a contribution of NK cells to IN1 and IN2 architectures with MAIT cell contribution to IN1 and IN3.

Taken together, our analysis revealed three IN types present to varying degrees in the HCC TME that are characterised by distinct immune cell types and cellular interaction patterns pointing to the existence of active and suppressive immune environments in human HCC.

### Immune neighbourhood architecture is linked to CD8 T-cell infiltration and differential compartmentalisation

**Figure 3:**
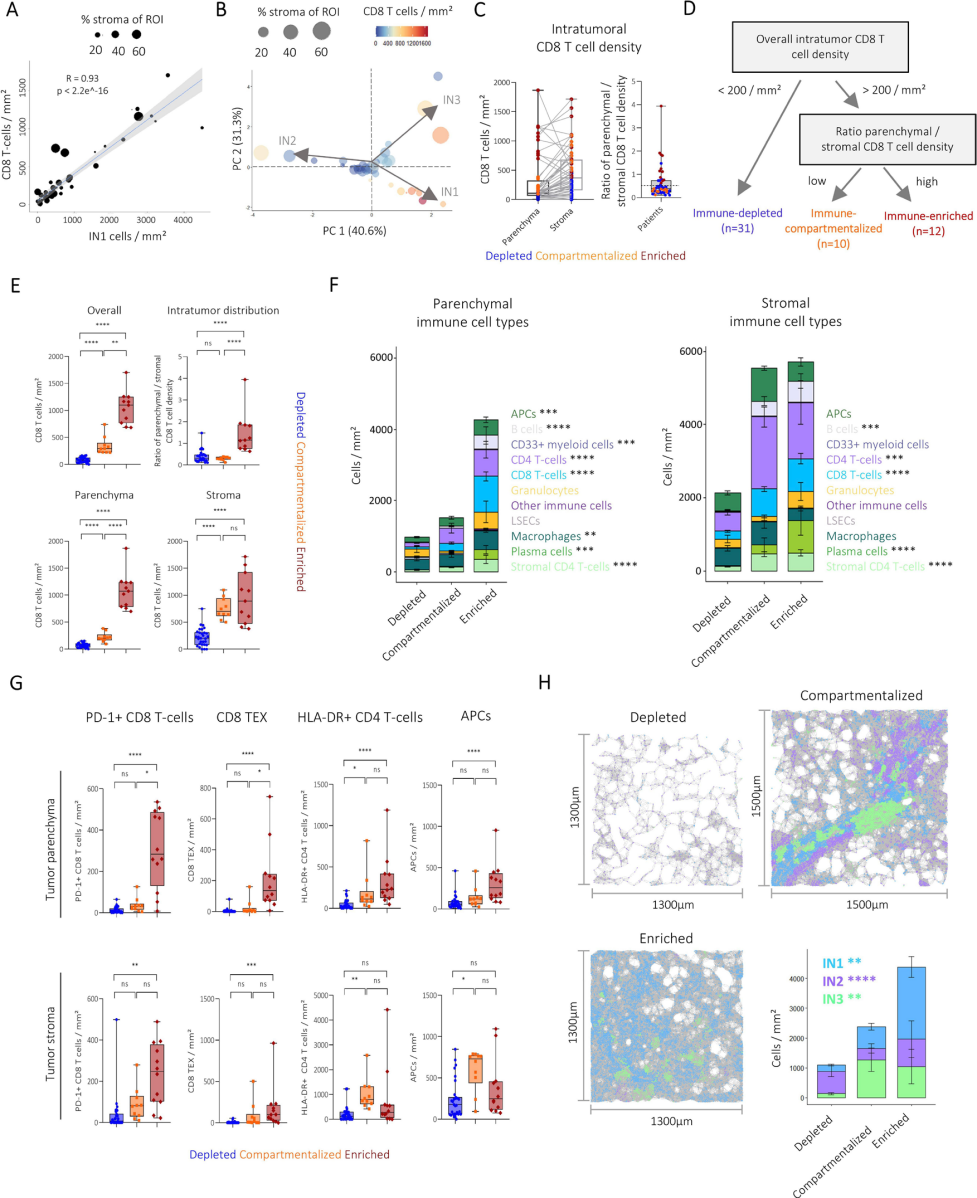
The amount and distribution of CD8 T cells define HCC spatial immune types with distinct immune microenvironments. (**A**) Pearson correlation of IN1 cell and CD8 T cell densities. Each dot represents one patient’s tumour ROI. Dot size indicates the percentage covered by stroma. (**B**) Principal component analysis based on IN density. Each dot represents one patient’s tumour ROI. Dot size indicates the percentage covered by stroma and colour the overall CD8 T cell density. Arrows visualise contribution of each parameter to principal components 1 and 2. (**C**) Boxplot showing CD8 T cell density in the tumour parenchyma and stroma (left) and the ratio of parenchymal to stromal CD8 T cell density. Dots represent patients and are coloured by spatial immunotype. (**D**) Schematic visualisation of patient classification into spatial immunotypes based on intratumoural CD8 T cell infiltration and distribution. (**E**) Boxplots showing the comparison of overall (top left), parenchymal (bottom left), stromal (bottom right) and the ratio between parenchymal and stromal CD8 T cell density (top right) between spatial immunotypes. (**F**) Stacked bar plots visualising mean immune cell type composition in the tumour parenchyma (left) and tumour stroma (right) of spatial immunotypes. Error bars indicate SE. Comparisons were performed using Kruskal-Wallis tests and Bonferroni corrected for multiple comparisons. (**G**) Boxplots showing immune subset densities in the tumour parenchyma (top) and stroma (bottom) between spatial immune types. Each dot represents a patient, pairwise tests were performed using Mann-Whitney U tests and Bonferroni corrected for multiple comparisons. (**H**) Example images showing cells coloured by immune neighbourhoods with their interactions (grey lines) for a depleted (top left), compartmentalised (top right) and enriched (bottom left) patient. Stacked bar plots showing mean immune neighbourhood density with error bars for each spatial immune type. Error bars indicate SE. Immune neighbourhood frequencies were compared using Kruskal-Wallis tests and Bonferroni corrected for multiple comparisons. ns, not significant, *p<0.05, **p<0.01, ***p<0.001, ****p<0.0001. HCC, hepatocellular carcinoma; IN, immune neighbourhood; ROI, region of interest.

We sought to deconvolute parameters that capture the heterogeneity of immune architecture. CD8 T cell abundance correlated significantly with the abundance of IN1 neighbourhood, however, there were also some HCC tumours in which this correlation was not observed (figure 3A). Interestingly, in those few HCCs, we noted a particularly high intratumoural stroma fraction. Principal component analysis (PCA) based on the IN density of tumour ROIs revealed three major axes of variation and highlighted that one axis of variation correlated with tumour infiltrating CD8 T-cell density except for the tumours with highest intratumoural stroma content (figure 3B). CD8 T-cell density within the tumour stroma was highly variable, and high tumour parenchymal CD8 T cell density was linked to higher CD8 T cell numbers in the stroma. However, we also noted a group of HCCs with a strong reduction in T-cell numbers between the tumour stroma and parenchyma and again others had low densities in both compartments (figure 3C, online supplemental figure 1). This analysis indicated that features of CD8 T-cell infiltration and intratumoural distribution between parenchyma and stroma represent major correlates of variation of the spatial immune architecture.

### Establishment of a spatial immune classification for the HCC TME

For translation of spatial immunotypes into clinical diagnostic practice, a reductive approach would be preferable. Based on our findings above, we, therefore, developed a spatial immune classification taking into account the amount and distribution of infiltrating CD8 T-cells. Reflecting the quantity and ability of CD8 T-cells to infiltrate the tumour parenchyma, we grouped HCC specimen with a CD8 T-cell density below 200 per mm^2^ as immune-depleted (58.5%) while HCCs with a CD8 T-cell density above this cut-off were further classified into compartmentalised (18.9%) or immune-enriched (22.6%) based on the ratio between parenchymal and stromal CD8 T cell densities below or above 0.5, respectively, as illustrated in figure 3D. Analysis of CD8 T-cell densities revealed the highest overall CD8 T-cell numbers in immune-enriched HCCs, whereas compartmentalised HCCs showed intermediate amounts and lowest CD8 T-cell frequencies were found in depleted samples, as expected. Interestingly, while the stromal CD8 T-cell density did not differ between enriched and compartmentalised HCCs, the difference in quantity was due to significant infiltration of CD8 T-cells into the tumour parenchyma in these groups that both harboured high levels of immune cells, a feature only revealed by integrating the spatial microenvironment component of the analysis (figure 3E).

Next, we tested whether this immunotype classification would reveal other distinct components and cellular interactions of the tumour immune microenvironment. Therefore, we compared the abundance of other immune cell types and subsets in the tumour parenchyma and stroma and their interactions. We observed significant differences between spatial immunotypes in both compartments (figure 3F,G, online supplemental figure 3). In the tumour parenchyma, overall immune infiltration was significantly reduced in patients with depleted and compartmentalised immunotype compared with immune-enriched patients. These differences were largely due to the lower numbers of CD8 T-cells, as expected, but also CD4 T-cells, B cells, plasma cells, macrophages and APCs (figure 3F). In the tumour stroma, compartmentalised and enriched immunotypes exhibited a comparable amount of immune cell infiltration. However, we noted several qualitative differences in the stroma of the enriched immunotype, such as a skewing towards higher plasma cell infiltration and Treg infiltration in the parenchyma while CD4 T-cell and APC infiltration tended to increase in the stroma of compartmentalised HCCs (figure 3F, online supplemental figure 3B). Of note, we also found that CD8 T-cells in the tumour parenchyma and stroma of the immune-enriched immunotype had significantly upregulated PD-1 and other inhibitory receptors (figure 3G) and interacted with PD-L1+APCs and macrophages (online supplemental figure 4A), suggesting that they could be targeted by current ICI therapies. Moreover, TRMs were enriched in the parenchyma of enriched patients (online supplemental figure 3A). In contrast, the compartmentalised immunotype was characterised by a significant increase of HLA-DR+CD4 T-cells and APCs, and a tendency towards higher densities of CD204+APCs in the stroma only. Treg and Tfh cells interacted with APCs, especially HLA-DR+CD4 T-cells (online supplemental figure 3B). The depleted immunotype showed the lowest density for all immune subsets (figure 3F–G, online supplemental figure 3) and an overall scarcity of direct immune cell interactions (online supplemental figure 5A) with a trend towards elevated frequencies of CD204+APCs in both compartments (online supplemental figure 5B). These analyses demonstrate that the spatial immunotypes are characterised by qualitative and quantitative differences in terms of immune cell composition and interactions, as well as contributions of the INs (figure 3H).

In sum, we developed a simplified spatial immunotype classification for the HCC TME based on CD8 T-cell infiltration and microanatomic distribution that reflected major axes of variation of tumour-immune architecture. Notably, this classification is also connected to different types of immune cells with varying expression of immune checkpoints.

### Comparison of the immune architecture in the tumour stroma and the invasive margin

The spatial immunotype classification developed above was based on the analysis of the intratumour immune architecture. Since it revealed a major role for both the extent of immune infiltration and the intratumour stroma compartment, we wondered how these findings were connected to the tumour margins, the invasive area expected to regulate immune cell infiltration.

**Figure 4:**
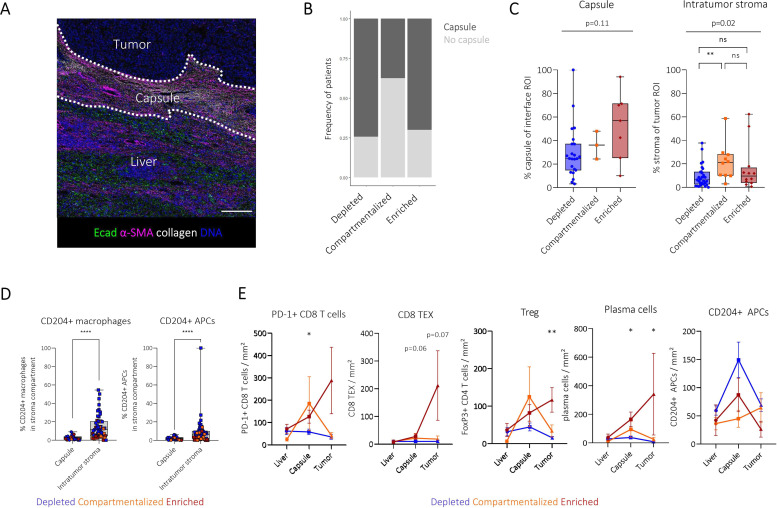
Deep spatial profiling of the HCC tumour interface. (**A**) Example image visualising interface compartments: adjacent liver, stroma capsule and tumour. Scale bar indicates 200 µm. (**B**) Stacked bar plot indicating the frequency of patients with tumour capsule formation between spatial immune types. (**C**) Boxplot comparing percentage of pixels belonging to the capsule in the interface ROI (left) and intratumour stroma of the tumour ROI (right) inferred by interface and stroma maps, respectively. (**D**) Frequency of CD204+ macrophages and APCs compared between the capsule and intratumour stroma. Each dot represents a patient and is coloured by spatial immune type. (**E**) Connected dot plots visualising mean and SE of respective immune subset densities for each spatial immunotype. Kruskal-Wallis tests were used to test for significant differences between spatial immune types within each interface compartment. *p<0.05, **p<0.01, ****p<0.0001. APC, antigen-presenting cell; HCC, hepatocellular carcinoma; ns, not significant; ROI, region of interest.

We generated spatial maps at the tumour interface to identify adjacent liver, capsule and tumour niches (figure 4A). A tumour capsule was observed in the majority of our patients (61.1%). Of note, the compartmentalised group showed the lowest prevalence of a tumour capsule (figure 4B). In contrast, compartmentalised HCCs revealed increased amounts of intratumour stroma (figure 4C). Moreover, we found that M2-like myeloid cells were significantly increased in the intratumour stroma compartment compared with the tumour capsule, suggesting different immunosuppressive environments in the intratumour stroma compared with the tumour capsule (figure 4D).

Since the tumour capsule is considered a barrier between the tumour and adjacent liver, we wondered if capsular immune cell infiltration was altered and whether there were differential dynamics between the spatial immunotypes across interface compartments. Interestingly, we identified an increase in PD-1+CD8 T-cells, CD8 TEX, Tregs and plasma cells from the adjacent liver, through the capsule to the tumour in enriched immunotype patients only (figure 4E). In contrast, in the depleted immunotype, we found an increase in immunosuppressive CD204+APCs in the capsule while the density in the tumour compartment was similar between depleted and compartmentalised patients (figure 4E). These findings point towards differential roles of the tumour capsule and its potential immunosuppressive features between spatial immunotypes.

### Impact of genetic alterations and HCC risk factors on the immunotypes

**Figure 5:**
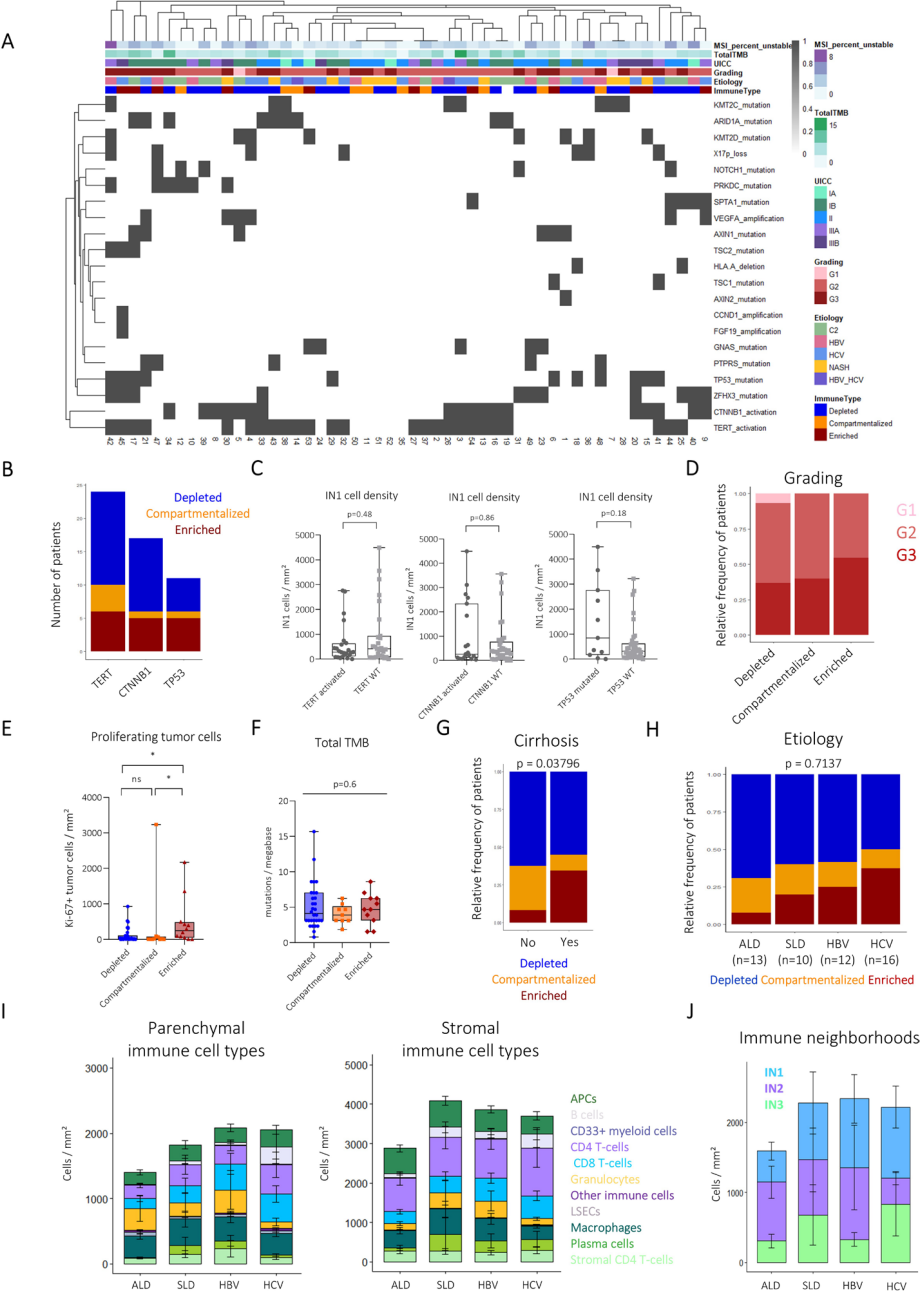
Tumour molecular profiles and underlying HCC aetiology do not dictate immune organisation in the HCC TME. (**A**) Heatmap showing frequent molecular alterations assessed by the TSO-500 panel of each patient. Annotations are based on clinical and immune features. (**B**) Stacked bar plots visualising the number of patients belonging to each spatial immune type of the patients with the indicated mutations. (**C**) Boxplots comparing the density of IN1 cells between patients with and without respective mutations. Statistical significance was assessed by Mann-Whitney tests. (**D**) Stacked bar plot showing the relative frequency of tumour grades G1–3 within each spatial immune type. (**E**) Boxplot comparing the density of Ki-67+ tumour cells between spatial immune types. Pairwise statistical differences were assessed by Mann-Whitney U tests. (**F**) Boxplots comparing total tumour mutational burden (TMB) between spatial immune types. P value was inferred by Kruskal-Wallis test. (**G**) Stacked bar plot comparing immunotype contribution to patients with and without pathologically defined cirrhosis in the surrounding liver tissue. χ^2^ test was applied to assess statistical significance. (**H**) Stacked bar plots showing the contribution of spatial immune types to patients grouped by underlying liver disease. χ^2^ test was applied to assess statistical significance. (**I**) Stacked bar plots visualising mean cell type densities in the tumour parenchyma (left) and stroma (right) between patients with differing HCC aetiologies. Error bars indicate SE. Kruskal-Wallis tests were used to test for overall significant differences. Mann-Whitney U tests were used to assess pairwise statistical differences and p values were Bonferroni-corrected for multiple comparisons. (**J**) Stacked barplots visualising mean immune neighbourhood densities in the tumour ROIs of patients with differing HCC aetiologies. Error bars indicate SE. Kruskal-Wallis tests were used to test for overall significant differences. Mann-Whitney U tests were used to assess pairwise statistical differences and p values were Bonferroni-corrected for multiple comparisons. *p<0.05. ALD, alcohol-related liver disease; HBV, hepatitis B virus; HCC, hepatocellular carcinoma; HCV, hepatitis C virus; ROI, region of interest; SLD, steatotic liver disease; TME, tumour microenvironment.

We next asked whether specific genetic alterations are connected to the organisation of the TME. Tumour samples were analysed for genetic variants, copy number variations, structural alterations and overall tumour mutational burden (TMB) and microsatellite instability status. As expected for HCC, our cohort was heterogeneous regarding the genetic landscapes with only a few alterations being present in more than 20% of patients (figure 5A). As expected, mutations in the *TERT-*promoter, and the *CTNNB1* and *TP53* genes represented the most frequent genetic alterations. *CTNNB1*-activated tumours were found within all spatial immunotypes (figure 5B). Despite a skewing of *TP53*-mutated tumours towards immunotypes with enhanced T-cell infiltration and the IN1 neighbourhood, there was overall no clear link between genetic alterations and the identified immunotypes (figure 5B,C). The enriched immunotype was also characterised by a higher frequency of Ki67+proliferating tumour cells, in line with a trend to more aggressive tumour grading but did not show a significantly higher TMB (figure 5D–F). There was a bias towards the enriched immunotype in patients with cirrhotic liver tissue surrounding the tumour as defined by a pathologist (figure 5G).

The aetiology of underlying liver disease as the major driver of HCC development may elicit different immune responses and impact the organisation of the TME.^15^ Nevertheless, in our cohort, we did not observe a significant connection between the underlying risk factors of HCC development and spatial immune types, immune cell type composition and INs (figure 5H–J). However, we observed a trend towards an increased IN1 neighbourhood and enriched immunotype contribution in patients with chronic viral hepatitis. In sum, mutational profiles as well as tumour aetiology per se do not predict the spatial immunotypes. These results suggest that the organisation of the HCC immune architecture in the TME is not primarily determined by tumour genetics or aetiology, highlighting that spatial immunotypes provide an independent level of information regarding tumour biology.

### Validation of the spatial classification approach in a distinct cohort

Next, we validated our approach to classify the HCC tumour immune microenvironment on a spatially resolved, single-cell level using CD8 T-cell infiltration features in another set of HCC samples on both a high-dimensional and immunotype level and correlated the identified immunotypes with response and survival under ICI therapy.

**Figure 6:**
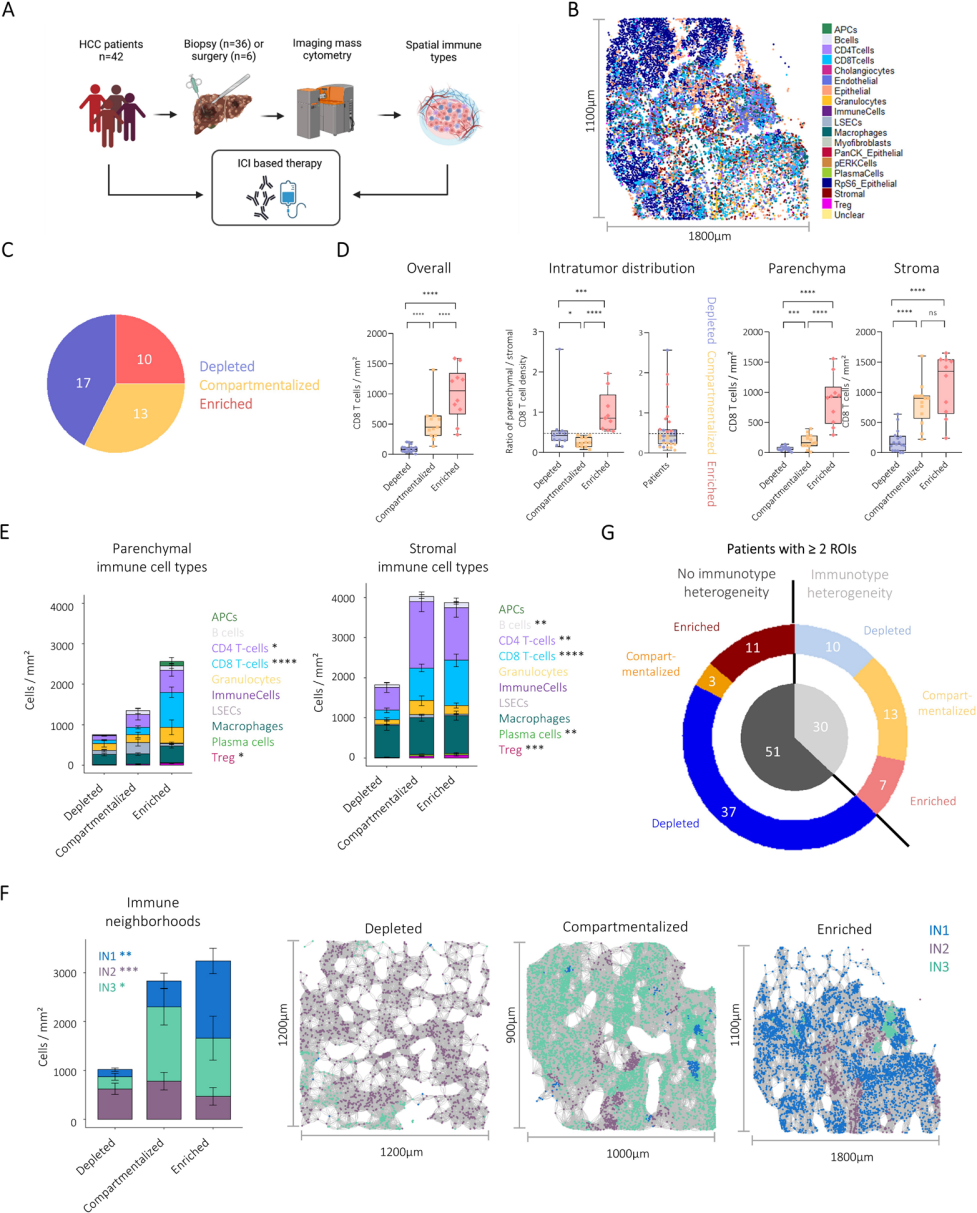
Validation of the immune network analysis and spatial immune classification in an independent cohort. (**A**) Illustration of the workflow used to assess the connection of spatial immune types and response to ICI-based therapy in HCC patients. (**B**) Identified cell types visualised in one HCC patient from the validation cohort. (**C**) Pie chart showing the number of patients belonging to each spatial immune type in the ICI cohort. (**D**) Boxplots comparing CD8 T cell infiltration patterns in microanatomical compartments between spatial immune types. (**E**) Stacked bar plots visualising mean immune cell type composition in the tumour parenchyma (left) and stroma (right) of spatial immune types. Error bars indicate SE. Comparisons were performed using Kruskal-Wallis tests and Bonferroni corrected for multiple comparisons. (**F**) Example images of immune neighbourhoods in a depleted (left), compartmentalised (middle) and enriched (right) patient. Stacked bar plots visualising mean immune neighbourhood density of spatial immune types. Error bars indicate SE. Comparisons were performed on IN frequencies using Kruskal-Wallis tests and Bonferroni corrected for multiple comparisons. (**G**) Plot visualising the number of patients with and without intratumour heterogeneity (inner pie chart) and the contribution of spatial immunotypes (outer donut chart) if two or more ROIs were acquired from patients of the discovery and validation cohort together. *p<0.05, **p<0.01, ***p<0.001, ****p<0.0001. HCC, hepatocellular carcinoma; ICI, immune checkpoint inhibitor; ns, not significant; ROIs, regions of interest.

We analysed an independent cohort of 42 HCC patients who had biopsies (n=36) or surgical samples (n=6) available prior to ICI-based therapy (figure 6A). Cell clustering and immune network analysis based on spatial interactions revealed similar cell types and INs compared with the discovery cohort (figure 6B, online supplemental figure 6A, B). Next, spatial immune types were identified based on CD8 T-cell infiltration and distribution between the parenchymal and stromal compartments as in our discovery cohort. We then asked if our approach determined in resected tissue was applicable to biopsy specimen as well. 2/36 biopsies had insufficient material for a spatial immune classification. In all other patients (94,4%, n=34/36 biopsies), the spatial immune classification was successfully reproduced. We identified (n=17) immune-depleted, (n=13) immune-compartmentalise and (n=10) immune-enriched HCCs in this cohort (figure 6C). As expected, CD8 T-cell infiltration patterns were comparable between the validation and the discovery cohort (figure 6D, figure 4). Additionally, infiltration with other immune cell types differed between spatial immune types in both compartments, as expected from observations in the discovery cohort (figure 6E). The INs also showed similar correlations to spatial immunotypes, with the CD8 T-cell hub (IN1) increased in enriched HCCs, the CD4/B cell hub (IN3) in compartmentalised HCCs and the myeloid hub (IN2) in depleted HCCs (figure 6F).

### Intratumour heterogeneity and HCC immune architecture

We here assigned immunotypes to individual patients’ ROIs. HCC is known to have significant intratumour heterogeneity. We found that some patients in the validation cohort also had intratumour heterogeneity with respect to immunotypes in different ROIs (online supplemental figure 6C). In biopsies, a consensus classification based on the most dominant immunotype observed was used. However, to formally investigate the impact of HCC heterogeneity on immune architecture, we also acquired a second ROI in a different location from 52 patients of the discovery cohort. Across both cohorts, immunotype heterogeneity was observed in about 1/3 of patients (figure 6G). Patients with one compartmentalised ROI were disproportionately affected by intratumour heterogeneity (figure 6G, online supplemental figure 6C, D). The observed heterogeneity was not due to sampling of limited stroma since the exclusion of ROIs with less than 5% stroma or parenchyma areas did not reduce the percentage of patients with immunotype heterogeneity (online supplemental figure 6E). In most patients with immunotype heterogeneity the overall immune cell density differed between the ROIs, however, the relative cellular immune architecture as described by the identified INs was less affected by tumour heterogeneity (online supplemental figure 6F).

### The spatial immune classification discriminates distinct outcome of checkpoint therapy

**Figure 7:**
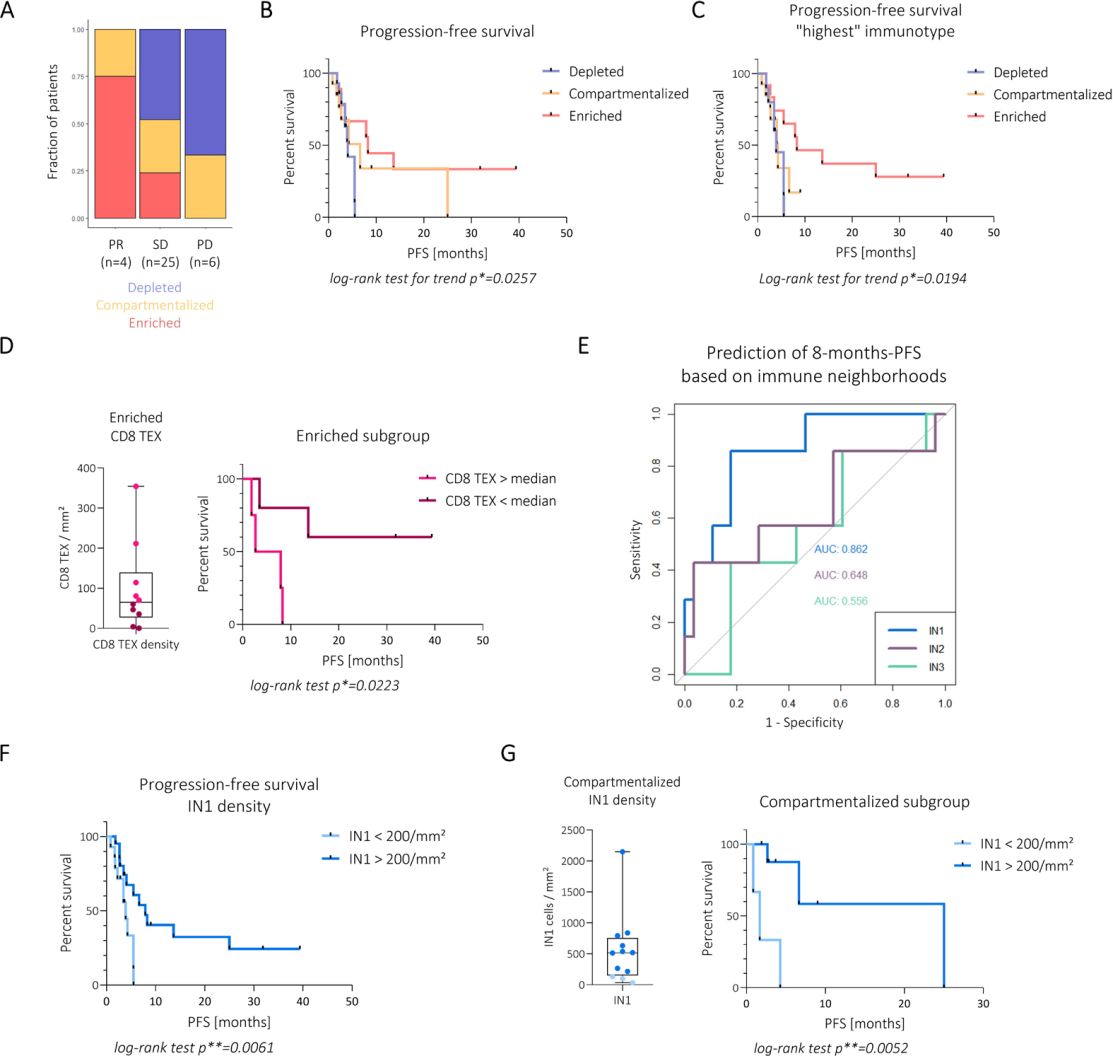
The spatial immune architecture of HCC patients correlates with immune checkpoint inhibitor therapy outcome. (**A**) Patients are grouped by best response assessed by RECIST V.1.1 into partial response (PR), stable disease (SD) and progressive disease (PD). Stacked bar plots show contribution of spatial immune types. (**B**) Kaplan-Meier curves of progression-free survival (PFS) of patients belonging to spatial immune type groups. Statistical significance was assessed by log-rank test for trend. (**C**) Kaplan-Meier survival curve comparing PFS of spatial immunotypes if patients were assigned the highest immunotype present in at least one ROI. Log-rank test for trend was used to assess statistical significance. (**D**) Left: Boxplot showing CD8 TEX density in enriched patients. Each dot represents a patient and is coloured by CD8 TEX density above or below the median. Right: Kaplan-Meier curves comparing PFS of CD8 TEX high and low patients. Log-rank test was used to assess statistical significance. (**E**) ROC curves comparing the sensitivity and specificity for the prediction of 8 months PFS of immune neighbourhood densities. (**F**) Kaplan-Meier curves comparing PFS of IN1 high (>200 cells/mm²) and low (<200 cells/mm²) patients. Log-rank test was used to assess statistical significance. (**G**) Left: Boxplot showing IN1 density in compartmentalised patients. Each dot represents a patient and is coloured by IN1 density above or below 200 cells/mm². Right: Kaplan-Meier curves comparing PFS of compartmentalised patients with IN1 high and low density. Log-rank test was used to assess statistical significance. *p<0.05, **p<0.01. AUC, area under the curve; ns, not significant; RC, receiver operaror characteristic; ROI, region of interest.

We next analysed whether the spatial immune classification can predict ICI therapy outcome. First, we found that spatial immunotypes were associated with the best overall response defined by RECIST V.1.1 criteria (figure 7A). Specifically, immune-enriched and compartmentalised immunotypes were found in patients with an objective response. In all patients with the enriched immunotype ICI therapy led to at least stable disease. Importantly, a comparison of progression-free survival (PFS) between spatial immune types revealed significantly prolonged PFS in immune-enriched patients with a median PFS of 8.3 months vs 6.6 months in compartmentalised and 4.1 months in depleted patients (figure 7B, online supplemental figure 7A). Correlation analyses with clinical variables highlighted the enriched and depleted immunotypes as significant correlates of PFS and overall survival (OS) (online supplemental figure 7B). We asked if classifying patients who displayed intratumour heterogeneity in different ROIs according to the ‘highest’ immunotype present in at least one ROI (assuming a hierarchy from enriched over compartmentalised over depleted) would be associated with different outcomes compared with classifying patients according to the most dominant immunotype. This ‘highest-immunotype’ classification approach led to a change in patient classification in six patients compared with the consensus model. Importantly, it revealed that the PFS benefit is even more pronounced for patients who had at least one enriched ROI whereas compartmentalised and depleted patients had similarly reduced PFS (figure 7C). These data demonstrate a clear link between spatial immunotypes and therapy outcomes and highlight them as potential pretherapeutic biomarkers.

We next asked if qualitative T-cell features could provide further insights within the defined immunotypes, such as in the subgroup of immune-enriched patients with treatment resistance. In our previous work, we found that the relative exhaustion of the CD8 T-cell response was a predictor of survival in HCC.^12^ Here, we noticed a significant enrichment of CD8 TEX and Tfh cells in enriched patients with reduced PFS (online supplemental figure 7C). As shown in figure 7D, patients with reduced numbers of exhausted T-cells within the enriched immunotype had the longest long-term survival.

To re-evaluate the cutoffs for the classification that were initially derived from the data from the discovery cohort only, we now took advantage of the full dataset from the discovery and validation cohort. A CD8 T-cell density cutoff of 200 per mm2 and a parenchymal to stromal ratio cutoff of 0.6 reflected the data distribution best (online supplemental figure 7D,E). An analysis based on those cutoffs more clearly separated the patient outcomes in the ICI therapy-treated cohort and, using the “highest-immunotype” approach for patients with ITH, discriminated significantly different PFS and OS (online supplemental figure 7F,G).

We next examined if the INs per se could be used to differentially predict outcomes between the immune-enriched and immune-compartmentalised groups. Of note, we observed that IN1, the predominant IN of enriched patients, well-predicted PFS at 8 months (figure 7E) and a high density of IN1 was able to predict prolonged PFS in the overall patient cohort (figure 7F). Interestingly, in compartmentalised patients, IN1 density (but not IN2, IN3) was able to discriminate between patients with short and prolonged survival (figure 7G).

In sum, these results demonstrate a clear link between the spatial immunotypes and therapy outcomes of ICI therapy. In addition to the broad immunotypes, specific features of the spatial immune architecture, such as enrichment for IN1 and reduced T-cell exhaustion have the potential to further improve predictive accuracy.

## Discussion

Here, we mapped spatial immunotypes of HCC that predict for outcome from immunotherapy by applying deep spatial single-cell level profiling and bioinformatic deconvolution of the immune architecture of the HCC TME. Three major patterns of immune cell infiltration were observed that were defined by differing amounts and distributions of infiltrating CD8 T-cells between the tumour parenchyma and stroma: an immune-depleted, a compartmentalised and an immune-enriched immunotype. They exhibited differential contribution of adaptive and innate immune cell subsets and immune checkpoints. The immunotypes were defined in a discovery cohort well characterised for tumour aetiology and tumour mutational profile and validated in a cohort of patients who was treated with ICI therapy. Our work indicates that the spatial immunotypes are suitable biomarkers to predict ICI therapy outcome in HCC refining predictive approaches based solely on CD8 T-cell quantity.

Spatial immunotypes are now recognised as correlates of successful checkpoint therapy in many cancers.^1^ Preclinical and clinical models are rich in descriptions of ‘immune-desert‘, ‘T-cell cold’, ‘immune excluded’, ‘immune-active’, ‘inflamed’, ‘T-cell hot’ or ‘T-cell exhausted’ phenotypes, however, they remain rather conceptually defined.^25^ In this work, we provide a precise and translationally applicable definition of three relevant HCC immunotypes. Interestingly, extensive prior work on the molecular and histological heterogeneity of HCC has revealed a larger number of molecular subtypes, which include an ‘immune-active group’, however, these molecular classifications did not directly translate into predictive categories for immunotherapy approaches.^1726^,^28^ Of note, these approaches did not take into account precise immune cell composition and spatial immune architecture. In our work, CD8 T-cell infiltration and distribution in tumour microenvironmental niches were identified as a major determinant of variation across HCC samples.

Our analysis included tumours with a large number of molecular alterations identified through mutation profiling and included patients with varying disease aetiology (including steatotic liver disease (SLD) and viral hepatitis), arguing against a major selection bias in our cohort. Despite the potential connection of tumour aetiology with specific immune reactions in HCC, in particular, between metabolic liver disease-associated HCC and viral hepatitis-associated HCC,^15 29 30^ we did not find a strong correlation of the identified immunotypes with aetiology. It is, therefore, tempting to speculate that the spatial immunotypes already reflect a higher-level integration of immunogenic tumour–host interactions and thus are a direct correlate of the outcome of immunotherapy. This notion is supported by a molecular analysis of biomarkers in the IMbrave-150 trial, in which pre-existing immunity including CD8 T-cell density was associated with better clinical outcomes of the combination therapy vs TKI.^11^ Moreover, recent spatially resolved profiling of HCCs further highlighted specific immune cell types to correlate with therapy response, such as cellular triads consisting of PD-1+effector CD8 T-cells, CXCL13+T helper cells and dendritic cells.^31^

The immunological mechanisms underlying the immunotypes are likely to differ significantly. In the enriched immunotype, we observed a major accumulation of IN1 cells including CD8 T-cells that express the immune checkpoints PD-1, Lag-3 and Tim-3, as well as antigen-presenting cells expressing PD-L1. These findings suggest that T-cell exhaustion is a major mechanism of immune evasion in this immunotype but also provide a likely molecular explanation for why PD-1-based ICI therapies are effective. Nevertheless, our analysis also indicates that the degree of T-cell exhaustion in the enriched immunotype can be detrimental if overwhelming, as patients with a high proportion of exhausted T-cells expressing additional immune checkpoints (ie, Tim-3, Lag-3) have significantly worse survival after checkpoint therapy. This finding is in line with prior work describing an association of exhaustion with poor clinical outcome.^12 31^ This severely exhausted CD8 T-cell phenotype typical for terminally exhausted T-cells may not be reinvigorated by anti-PD-(L)1 treatment alone.^32 33^ These findings suggest that cotargeting of additional inhibitory receptors is a rational strategy that could enhance immune responses further in the immune-enriched group.

In contrast, the compartmentalised immunotype, a subgroup showing a relative retention of immune cells in the intratumoural stroma, is characterised by a tertiary lymphoid structure (TLS-)like IN with prominent interactions among antigen presenting cells (HLA-DR+CD4 T-cells and B cells) suggesting ongoing immune cell recruitment, but prevention of effective T-cell egress. In line with this notion, M2-like CD204+APCs and Tregs are enriched in the tumour stroma. Interestingly, recent work highlighted interactions between PD-L1+tumour-associated macrophages (TAMs) and non-conventional (MAIT) T-cells in HCC mediating immune exclusion.^34^ Thus, in the compartmentalised immunotype, myeloid-directed or stroma-directed therapies might represent rational combination therapies for current immune checkpoint therapies. It is possible that the add-on effect of bevacizumab (anti-VEGF) in combination with ICIs may be due to modification of the tumour stroma. Indeed, atezolizumab plus bevacizumab combination therapy was connected to higher efficacy in TMEs with high infiltration of Tregs and myeloid cells or elevated density of CD31+vessels compared with atezolizumab monotherapy.^11^

In the immune-depleted group, immune cell interactions are scarce and a myeloid IN with prominent interactions of immunosuppressive CD204+macrophages and APCs characterise the residual immune architecture. The low quantity of and interactions among antigen-presenting cells (APCs, macrophages, B cells, HLA-DR+CD4 T-cells) and activated CD8 T-cells in both microenvironmental compartments of the TME suggests a defective antitumour immune response with reduced antigen recognition. Potentially, locally disruptive therapies aimed at enhancing immune cell priming^35^ may synergise with immunotherapy in particular in the depleted immunotype.

Classification of patients according to tumour-immune architecture using detailed networks of immune cell types and the three dominant INs is preferable for precise characterisation but requires high-dimensional single-cell analysis and may thus not be a suitable approach for resource-limited settings. Importantly, the simplified classification strategy based on CD8+T cell densities and their intratumoural distribution recapitulates major features of immune architecture and can predict patient outcomes. Intratumour immunotype heterogeneity, that is, distinct spatial organisation of the immune response in different areas of the same tumour, may occur in a proportion of patients and should be taken into consideration for application of immunotype classification in clinical practice. In patients with intratumoural heterogeneity (ie, in which individual analysis of different regions of the tumour would rate them as differing immunotypes) two approaches for patient classification appear practicable—classification according to the most representative immunotype (reflecting the largest area of tumour analysed) or a ‘highest immunotype’ classification (enriched>compartmentalised>depleted). While the ‘representative’ approach is likely more reflective of the total immune architecture, interestingly, classifying patients according to the ‘highest’ immunotype category present in the tumour regions most clearly discriminated patients with the longest survival after immunotherapy. Our data indicate that even if only one tumour region exhibits the enriched immunotype, it could confer a survival advantage under immunotherapy.

In sum, our high-dimensional spatial single-cell profiling approach with immune architecture analysis revealed three major immunotypes of HCC that are correlated to the response to ICI therapy. We specifically developed a simplified approach for this classification based on the CD8 T-cell infiltration and stromal/parenchymal distribution that may facilitate broad utilisation of the novel classification in clinical practice, allowing for better patient stratification for current immunotherapies. The distinct organisation of the TMEs within the three spatial immune types suggest differential demands for combinatorial therapeutic strategies. Inclusion of the immunotypes into prospective clinical trials will be required to validate the spatial immune classification as a biomarker for the prediction of ICI therapy response and selection of coregulatory therapies.

## Methods

### Study cohort

For IMC analysis, formalin-fixed, paraffin-embedded (FFPE) samples from n=54 HCC patients were obtained after surgery as a discovery cohort at the Institute of Pathology of the University Hospital Heidelberg, Germany (online supplemental table 2). For independent validation and correlation with ICI therapy outcome, FFPE sections from a cohort of n=42 HCC patients undergoing bioptic sampling (n=36) or surgical resection (n=6) prior to ICI therapy were recruited as in.^12^ Patients with Child-Pugh score ≥7 (n=5) were excluded from survival analyses to avoid bias due to reduced liver function. Clinical data are outlined in online supplemental table 3. An additional cohort (n=5) of snap-frozen HCC tissues was recruited for analysis of innate-like immune cells.

### Next-generation sequencing

FFPE samples of the discovery cohort were subjected to genomic profiling at the Center of Molecular Pathology (MPZ), Institute of Pathology, University of Heidelberg, Germany using the TruSight Oncology 500 (TSO500, Illumina) panel as previously described.^36^

### IMC of FFPE tissue sections

A panel of n=40 metal-tagged antibodies focused on the detection of epitopes important for immune cell phenotyping and function in the HCC microenvironment was validated (online supplemental table 1). Antibodies were either bought preconjugated from Standard BioTools or coupled in-house using the Maxpar X8 labelling kit (Standard BioTools). Platinum conjugates were labelled as described.^37^ Antibody clones were titrated on liver, HCC and tonsil tissue to test for specificity and to determine optimal dilutions. FFPE sections were stained as described before.^12 38^ Briefly, the tissue slides were deparaffinised twice for 15 min in ROTIHistol (Carl Roth) and rehydrated in descending grades of ethanol. Antigen retrieval was performed with EnVision FLEX target retrieval solution (high pH) in a pressure cooker for 30 min at 95°C. Slides were cooled down, rinsed in TBS, the tissue was encircled with a hydrophobic pen and blocked with SuperBlock (TBS) for 45 min to decrease unspecific binding. Next, the slides were incubated with a mixture of metal-tagged antibodies overnight at 4°C in a wet chamber. Afterwards, slides were washed with TBS-T (TBS with 0.2% Tween-20), nuclear staining was performed and slides were finally rinsed in TBS and ddH_2_O, dried and stored until acquisition. ROIs were selected based on consecutive H&E stains in the tumour (with the goal to include tumour parenchyma and tumour stroma areas), the interface region and adjacent liver resulting in the acquisition of 110 ROIs in total with at least two ROIs per patient in the primary HCC cohort. In 52 patients with sufficient material one additional ROI of 1mm^2^ size in a different region on the same slide was acquired subsequently. In the ICI therapy cohort, up to four ROIs were selected per patient based on tissue size to acquire a minimum area of approximately 2.25 mm^2^ resulting in 86 ROIs. The tissue within the ROIs was ablated spot-by-spot by a laser with 200 Hz and the resulting ion clouds were transported in the mass cytometer for metal isotope quantification by time-of-fight resulting in multiplexed images of 1 µm^2^ resolution. Pseudocoloured composite images were visualised in MCDviewer (Standard BioTools) or ImageJ V.2.3.051. Two ROIs of the primary HCC cohort were excluded from further analysis due to staining artefacts.

### IMC of fresh-frozen tissue sections

A 32-antibody panel was developed, aimed at further dissecting innate-like immune cells (online supplemental table 1). Three µm snap-frozen tissue sections were mounted onto SuperFrost slides and stored at −80°C until stained for IMC imaging. The tissue slides were sequentially thawed at −20°C and 4°C for 1 hour each and subsequently fixed in 100% methanol at −20°C for 5 min. The tissue sections were encircled with a hydrophobic pen, incubated in SuperBlock (TBS) for 45 min at room temperature. Metal-conjugated antibody mixture was prepared in TBS at optimised dilutions, then used to stain tissue sections overnight at 4°C. Slides were rinsed twice in TBS-T and twice in TBS, each for 5 min and stained with Iridium Cell-ID intercalator for 30 min at room temperature. Subsequently, the tissue slides were rinsed three times in TBS for 5 min each. Finally, tissue sections were fixed with 8% glutaraldehyde for 30 s, rinsed three times in TBS for 5 min each and in ddH2O for five seconds, air-dried and stored until acquisition. IMC acquisition was performed using the Hyperion Imaging System (Standard BioTools). For each patient, 2–4 ROIs were acquired based on tissue size, covering a total minimum area of 1.96 mm^2^.

### Spillover compensation

The resulting .txt files from the acquisition were corrected for signal spillover using the CATALYST package in R as proposed by Chevrier *et al*.^39^

### Image processing and cell segmentation

Initial image processing and cell segmentation were performed using the steinbock pipeline.^40^ In short, stacked .tiff images were extracted from spillover-compensated .txt files and filtered for hot pixels. Single nuclei were detected based on Iridium and Histone H3 staining and expanded to cells based on membrane and cytoplasmic markers using the deep-learning segmentation algorithm Mesmer.^41^ The resulting cell masks were used to calculate mean marker expression and spatial features for each cell on the image while preserving X and Y coordinates and therefore a cell’s spatial context.

### Image normalisation

Image channels were normalised using PENGUIN—PErcentile Normalisation GUI Image deNoising,^42^ normalising signal intensities to values between 0 and 1, removing unspecific background signal and applying a percentile-based approach to reduce signal noise for each marker. Optimal percentiles and thresholds were chosen for each channel separately by manual inspection of the results on several images and applied to every ROI separately with the same values.

### Region maps

To distinguish parenchymal and stromal compartments, the random forest classifier ilastik V.1.3.3 was trained to classify pixels into parenchyma or stroma based on E-cadherin, CK7, SMA, collagen and CD34 staining. Binary masks were exported and converted into 16-bit .tiff images for further analysis using ImageJ V.2.3.051.

Maps to distinguish tumour, capsule and adjacent liver within the interface ROIs were created manually. A composite image was loaded into Gimp and different colours were used to mark the three compartments. The map was exported as a .Tiff file and converted into a 16-bit .Tiff image in Imagej V2.3.051.

### Single-cell and spatial analysis

Further analysis was performed in R V.4.2.2 based on the multiplexed imaging data analysis workflow proposed by Windhager *et al*^40^ using the R packages imcRtools and cytomapper.

A spatial experiment object was created by overlaying raw and normalised stacked .tiff images with the cell masks resulting in 1 274 949 single cells in the discovery cohort and 499 030 cells in the ICI cohort. Stroma and interface masks were integrated to map each cell’s location within the corresponding compartments. Raw channels were arcsinh transformed with a cofactor of 0.5 for further analysis.

Phenograph clustering was performed using the Rphenograph package with k=50 and based on normalised channels as listed in online supplemental table 1. For clustering of immune cells from the frozen tissue, gated CD45+cells were clustered based on normalised intensities of immune cell markers with k=20. Clusters with similar marker expression profiles were combined with cell types to account for overclustering.

### UMAP

A Uniform Manifold Approximation and Projection (UMAP) analysis was performed on all identified immune cells based on normalised immune markers using the runUMAP function from the scater package in R. Each cell was plotted in the resulting two-dimensional (2D) space and coloured by cell type.

### Identification of INs

Immune cell interactions were determined by constructing a k nearest neighbour graph using the buildSpatialGraph function from the imcRtools package. Each cell’s 40 closest neighbours on the image within a maximum radius of 75 µm were identified. Next, immune cells were clustered into INs based on their cell identity and spatial interactions using k-means clustering (kmeans function from the stats package in R) with three centres.

### Interaction analysis

Another neighbourhood graph was constructed by expanding each cell by 15 µm to account for closer cellular interactions. Touching cells after expansion were considered neighbours. Next, the function testInteractions using the ‘histocat’ method from the imcRtools package was used to test for interactions among immune cell subsets. Briefly, for each cell from cell subset A the number of neighbours of cell subset B was determined and divided by the number of cells from cell subset A that had a minimum of one neighbour from cell subset B for each ROI. The resulting interaction count was compared with the null distribution of spatial interactions of cell subset A and cell subset B derived by iterations of randomised cell localisations on the image. If the interaction count was within the highest or lowest 5% of the normal distribution it was considered a significant interaction or avoidance, respectively. The significance was tested for each ROI separately, and significant interactions and avoidances were summarised per spatial immune type for visualisation purposes. For close immune cell interactions within INs, the spatial experiment object was subset by IN and the same workflow as above was applied.

### Manual gating

Single-cells with their cell type identities and marker expression profiles were exported from R and loaded into OMIQ. Raw channels were arcsinh-transformed with a cofactor of 0.5 and hierarchically gated for specific markers to identify immune cell subsets starting from immune cell types. Thresholds were adapted for each ROI to account for sample-specific staining intensities. The gates were exported and reimported into R to transfer cell subset identities to the spatial experiment object.

### Principal component analysis

The PCA shown in figure 3B was conducted using the PCA function from the FactoMineR package based on IN density of each patient. Each patient’s location in 2D-space along principal components PC1 and PC2 was plotted using the ggscatter function from the ggplot2 package.

### Area calculation

The area covered by each biopsy or microenvironmental compartment was calculated in ImageJ by counting the pixels (one pixel=1 µm^2^) occupied by the corresponding tissue on the image or regional map.

### Cell density and frequency

Cell densities were calculated by dividing the number of cells by the ROI size or the area taken up by the biopsy or the microenvironmental compartment of interest. Frequencies were calculated as a subset of cells within a group of cells divided by the total number of cells from that group. If more than one ROI per patient existed, the mean was calculated for that patient. Cell densities and frequencies were plotted in GraphPad Prism V.8.0.1. Intratumoural CD8 T-cell distribution was computed as ((CD8 T-cell density in tumour parenchyma)/(CD8 T-cell density in tumour stroma)).

### Spatial immunotype classification

The overall, parenchymal and stromal CD8 T-cell densities as well as the ratio between parenchymal and stromal CD8 T-cell density were calculated for each ROI as described above. Cut-off values were determined according to the distribution of values in the discovery dataset and validated in the ICI therapy cohort. For a consensus classification of patients with immunotype heterogeneity between different ROIs, the most representative immunotype was determined as the most frequent immunotype among ROIs, or if equal numbers of different immunotype ROIs were present, the mean densities between those ROIs were calculated and the resulting immunotype assigned. An alternative approach was used for figure 7C—patients were classified according to the ‘highest’ immunotype, assuming a hierarchy from enriched>compartmentalised>depleted, that was present in at least one ROI.

### Statistical analysis

Statistical analyses were performed in R V.4.2.2 with the ggpubr package or GraphPad Prism V.8.0.1 using non-parametric tests since normal distribution of data was not assumed. For continuous variables if two groups with paired values were compared, Wilcoxon tests were applied. Unpaired values were compared using Mann-Whitney U tests. If three or more groups were compared, Kruskal-Wallis tests were used to determine overall significance of differences. Single comparisons were performed with Wilcoxon or Mann-Whitney U tests. Where applicable, p values were corrected for multiple comparisons using the Bonferroni correction method. Nominal variables were compared using χ^2^ tests (figure 5G,H, online supplemental table 4). Pearson correlation was used to assess the relation of CD8 T cell and IN IN1 cell densities shown in figure 3A. For the correlation matrix in online supplemental figure 7, correlations between two continuous variables were assessed by Spearman correlation. Correlations between two binary variables or one binary and one continuous variable were assessed by Wilcoxon tests. Benjamini-Hochberg correction was used to adjust p values for multiple comparisons.

For survival analysis, log-rank tests and log-rank tests for trend were used to assess statistically different survival between groups in the Kaplan-Meier survival curves in GraphPad Prism V.8.0.1.

### Boxplots

In each boxplot, boxes show median and IQR and whiskers extend from minimum to maximum values. Each dot represents one patient.

## supplementary material

10.1136/gutjnl-2024-332837 online supplemental file 1

10.1136/gutjnl-2024-332837 online supplemental table 1

10.1136/gutjnl-2024-332837 online supplemental table 2

10.1136/gutjnl-2024-332837 online supplemental table 3

10.1136/gutjnl-2024-332837 online supplemental table 4

10.1136/gutjnl-2024-332837 online supplemental figure 1

## Data Availability

Data are available in a public, open access repository. Data are available on reasonable request.
