# Supplementary material for: Spatial single-cell profiling and neighbourhood analysis reveal the determinants of immune architecture connected to checkpoint inhibitor therapy outcome in hepatocellular carcinoma
Source: Gut. 2024 Sep 30;74(3):e332837. doi: 10.1136/gutjnl-2024-332837 (PMC11874287; doi:10.1136/gutjnl-2024-332837)
Supplement: online supplemental table 1 [file gutjnl-74-3-s002.pdf]

Supplementary table 1: Imaging mass cytometry antibody panel

Metal	Target	Clone	Company	ID	Concentration [mg/ml]	Dilution 1/	Discovery cohort	ICI cohort	Frozen tissue coho	Phenograph clustering
89Y	CD45	D9M8I	CST	13917BF	0.3	100	X	X		X
89Y	CD45	HI30	BioLegend	304002	0.2	100			X	X
113In	VEGF	G153-694	BD	555036	0.2	100		X		
113In	CD68	KP1	BioLegend	91604	0.2	200	X			X
113In	CD68	KP1	BioLegend	91604	0.2	800			X	X
115In	HLA-DR	TAL-1B5	abcam	ab176408	0.2	400	X	X		X
115In	HLA-DR	TAL-1B5	abcam	ab176408	0.2	800			X	
127I	Höchst	/	MedChemExpress	158013-43-5	5.345	8000			X	
141Pr	SMA	1A4	fluidigm	3141017D	0.5	1600	X	X		X
141Pr	SMA	1A4	NOVUS	NBP2-34522	0.2	6400			X	X
142Nd	CD15	HI98	BioLegend	301902	0.2	800	X	X		X
142Nd	CD15	BA5b	Biolegend	302702	0.2	200			X	X
143Nd	LYVE1	EPR21857	abcam	ab232936	0.2	200	X	X	X	X
144Nd	CD69	EPR21814	abcam	ab234512	0.2	100	X	X		X
144Nd	CD69	FN50	Biolegend	310902	0.2	200			X	
145Nd	CD3	D7A6E	CST	85061BF	0.3	200	X	X		X
145Nd	CD206	C08C2	Biolegend	141702	0.2	200			X	
146Nd	Ki-67	B56	BD	556003	0.2	200	X	X		
146Nd	Ki-67	B56	BD	556003	0.2	400			X	
147Sm	CD163	EDhu-1	fluidigm	3147021D	0.5	1600	X	X	X	X
148Nd	Pan-keratin	C11	fluidigm	3148020D	0.5	200		X		X
148Nd	Pan-keratin	C11	Biolegend	628602	0.2	800			X	X
148Nd	TIGIT	TG1	Dianova	DIA-TG1-01MG	0.2	100	X			
149Sm	CXCR5	D6L3C	CST	72172BF	0.2	200	X	X		X
149Sm	Hepar	OCH1E5	abcam	ab190706	0.2	500			X	
150Nd	PD-L1	E1L3N	CST	13684S	0.2	50	X	X		X
151Eu	CXCR6	Poly	PA5-33462	WD3260492	0.2	100	X	X		
151Eu	CD161	HP-3G10	BioLegend	339902	0.2	50			X	X
152Sm	TCF-1	C63D9	CST	2203BF	0.2	100	X	X		X
152Sm	GranzymeB	CLB-GB11	NOVUS	NBP1-50071	0.2	50			X	
153Eu	TOX	NAN448B	abcam	ab237009	0.2	200	X	X		X
154Sm	Tim-3	D5D5R	fluidigm	3154024D	0.5	1600	X	X		X
154Sm	IL-18R	Polyclonal	R&D	AF840	0.2	100			X	
155Gd	FoxP3	236A/E7	Thermo fisher	14-4777-82	0.3	200	X	X		X
155Gd	FoxP3	236A/E7	Thermo fisher	14-4777-82	0.2	50			X	
156Gd	CD4	EPR6855	abcam	ab181724	0.2	400	X	X		X
157Gd	CX3CR1	8E10.D9	BioLegend	824001	0.2	200		X		
157Gd	Lag-3	EPR4392(2)	abcam	ab209740	0.2	800	X			X
158Gd	E-cadherin	24 E 10	fluidigm	3158029D	0.5	200	X	X		X
159Tb	CD68	KP1	fluidigm	3159035D	0.5	5000		X		X

159Tb	b-Catenin	D13A1	CST	33893	0.2	200	X			X
159Tb	PD-1	EH12.2H7	BioLegend	329902	0.2	50			X	
160Gd	T-bet	4B10	BioLegend	644802	0.2	100	X	X		X
160Gd	CD14	M5E2	BioLegend	301802	0.2	50			X	X
161Dy	CD20	H1	fluidigm	3161029D	0.5	800	X	X		X
161Dy	CD20	H1	BD	555677	0.2	50			X	X
162Dy	CD8a	C8/144B	BioLegend	372902	0.2	1600	X	X		X
162Dy	CD8a	C8/144B	BioLegend	372902	0.2	200			X	X
163Dy	Eomes	WD1928	Thermo fisher	14-4877-82	0.4	200	X	X		X
164Dy	Arginase-1	D4E3M	CST	93668BF	0.2	100		X		
164Dy	RpS6	A17020B	BioLegend	608602	0.2	1600	X			X
164Dy	GranzymeA	CB9	BioLegend	507202	0.2	100			X	
165Ho	PD-1	D4W2J	CST	86163BF	0.4	400	X	X		X
166Er	CD204	J5HTR3	invitrogen	14-9054-82	0.2	400	X	X		X
167Er	GranzymeB	EPR20129-217	fluidigm	3167021D	0.5	1600	X	X		X
167Er	CD38	HIT2	BioLegend	303502	0.2	200			X	X
168Er	CD39	EPR20627	abcam	ab236038	0.2	50	X	X		X
168Er	CD7	eBio124-1D1		14-0070-82	0.2	800			X	X
169Tm	Collagen I	Poly	fluidigm	3169023D	0.5	800	X	X		X
169Tm	Collagen I	Poly	Chemicon	AB758	0.2	8000			X	
170Er	CD103	SP301	abcam	ab245746	0.2	100	X	X		X
170Er	CD3	UCHT1	invitrogen	14-0038-82	0.2	400			X	X
171Yb	CD56	123C3	invitrogen	07-5603	0.2	200		X		X
171Yb	pERK1/2	D13.14.4E	fluidigm	3171021D	0.2	200	X			X
172Yb	CD38	EPR4106	abcam	ab176886	0.2	100	X	X		X
172Yb	CD11b	ICRF44	BioLegend	301337	0.2	100			X	X
173Yb	CD45RO	UCHL1	BioLegend	304202	0.2	800	X	X		X
173Yb	CD45RO	UCHL1	BioLegend	304202	0.2	200			X	X
174Yb	CD33	SP266	abcam	ab238784	0.2	100	X	X		X
174Yb	CD4	RPA-T4	BioLegend	300502	0.2	100			X	X
175Lu	CD34	EP373Y	abcam	ab198395	0.2	400	X	X		X
175Lu	CD34	581	BioLegend	343502	0.2	100			X	X
176Yb	CK7	RCK105	Origene	BM6003P	0.2	400	X	X		X
176Yb	CD56	NCAM16.2	BD	559043	0.2	200			X	X
194Pt	HH3	D1H2	CST	4499BF	0.2	200	X	X	X	
