# Supplementary material for: Spatial single-cell profiling and neighbourhood analysis reveal the determinants of immune architecture connected to checkpoint inhibitor therapy outcome in hepatocellular carcinoma
Source: Gut. 2024 Sep 30;74(3):e332837. doi: 10.1136/gutjnl-2024-332837 (PMC11874287; doi:10.1136/gutjnl-2024-332837)
Supplement: online supplemental table 2 [file gutjnl-74-3-s003.pdf]

Supplementary table 2: Discovery cohort

Patient ID	Age	Sex	Etiology	ImmuneType	TSO500	Tumor ROI	Second tumor ROI	Interface ROI	Adjacent ROI	Overall survival [months]	Censored [0=yes]	Cirrhosis (histological)	Grading (TMA)	Tu max size [cm]	nodules [n=]	Vascular invasion [1=yes]	UICC stage	R status
DC_01	55	m	ALD	Depleted	X	X	X	X		73	1	1	2	3	2	0	II	0
DC_02	70	m	HBV	Depleted	X	X		X		30	0	0	2	11	>3	1	IIIA	1
DC_03	70	m	HCV	Depleted	X	X	X	X		5	1	1	2	15	>3	1	IIIA	x
DC_04	67	f	HCV	Depleted	X	X	X	X		1	0	1	2	3	1	0	IB	0
DC_05	63	m	ALD	Depleted	X	X	X	X		51	1	1	1	2.7	1	0	IB	0
DC_06	63	m	HBV	Enriched	X	X	X		X	54	1	1	3	3.7	1	0	Ib	0
DC_07	41	f	SLD	Depleted	X	X	X		X	124	0	0	1	12	2	1	IIIA	0
DC_08	60	m	ALD	Depleted	X	X	X		X	29	1	0	2	7	>3	1	IIIA	x
DC_09	75	f	HCV	Enriched	X	X	X	X		56	0	1	2	5.5	>3	1	IIIA	0
DC_10	39	m	HBV	Depleted	X	X	X		X	3	1	0	2	11	3	1	IIIA	0
DC_11	60	m	SLD	Compartmentalized	X	X	X		X	53	0	0	3	7.5	1	1	II	0
DC_12	66	m	ALD	Compartmentalized	X	X	X	X		48	0	0	2	2.2	1	0	IB	0
DC_13	72	m	SLD	Compartmentalized	X	X	X		X	38	1	0	2	21	1	1	II	0
DC_14	51	m	HBV_HCV	Compartmentalized	X	X	X	X		101	1	1	2	2.5	1	1	II	0
DC_15	57	m	SLD	Enriched	X	X	X	X		31	1	1	3	4	2	1	IIIB	0
DC_16	74	m	ALD	Depleted	X	X	X		X	4	1	0	2	3.5	1	0	IB	0
DC_17	64	m	ALD	Enriched	X	X	X		X	33	0	1	3	4	1	0	IB	0
DC_18	38	f	HCV	Depleted	X	X	X		X	99	0	0	2	6.5	1	0	IB	0
DC_19	62	m	ALD	NA	X	excluded	X	X		58	0	1	2	2.3	1	0	IB	0
DC_20	58	m	HCV	Enriched	X	X			X	25	0	1	2	14	1	1	IIIB	0
DC_21	70	m	HCV	Depleted	X	X	X	X		88	0	0	3	4.6	1	0	IB	0
DC_22	71	m	HCV	Enriched		X	X		X	69	0	0	2	3.2	1	0	IB	0
DC_23	64	f	ALD	Compartmentalized	X	X	X		X	32	1	0	3	4	>3	1	II	0
DC_24	60	f	HCV	Depleted	X	X	X		X	5	0	1	2	5	>3	1	IIIB	0
DC_25	66	m	ALD	Depleted	X	X	X	X		65	0	1	2	5	1	1	II	0
DC_26	63	m	HBV_HCV	Depleted	X	X	X	X	X	20	0	1	2	5.5	>3	1	IIIA	0
DC_27	33	m	HBV	Enriched	X	X	X		X	3	0	0	3	10	1	1	IIIB	1
DC_28	68	m	SLD	Depleted	X	X	X	X		35	0	0	3	11	1	0	IB	0
DC_29	71	m	SLD	Depleted	X	X	X	X		2	0	0	3	10.5	1	1	IIIB	0
DC_30	67	m	SLD	Enriched	X	X	X		X	52	0	0	3	1.1	2	0	II	1
DC_31	75	m	ALD	Depleted	X	X	X		X	3	0	1	3	3	1	1	II	0
DC_32	79	m	HCV	Depleted	X	X	X	X		50	0	1	2	3.5	1	0	IB	0
DC_33	59	f	HCV	Depleted	X	X	X	X		2	0	0	3	6.5	1	1	II	0
DC_34	63	m	ALD	Depleted	X	X	X	X		54	0	0	3	3.2	1	0	IB	0
DC_35	61	f	HCV	Compartmentalized	X	X	X	X		53	0	0	2	2.2	1	1	II	0
DC_36	61	m	HBV	Depleted	X	X	X	X		53	0	1	2	6.5	1	1	II	0
DC_37	74	m	ALD	Compartmentalized	X	X	X	X		48	0	1	2	3.8	1	0	IB	0
DC_38	68	m	HCV	Compartmentalized	X	X	X	X		51	0	0	2	2	1	0	IA	0
DC_39	70	m	ALD	Depleted	X	X	X	X		10	0	1	2	1.6	1	0	IB	1
DC_40	67	m	HBV	Depleted	X	X	X	X		47	0	1	2	1.7	1	0	IA	0
DC_41	62	f	HCV	Depleted	X	X	X	X		78	1	1	3	6.2	>3	0	IIIA	0

m	HBV	Depleted	X	X	X	X		4	0	0	3	17.5	>3	1	IIIA	x
m	SLD	Depleted	X	X	X	X		46	0	1	3	3.8	3	0	II	0
m	ALD	Depleted	X	X	X	X		32	0	1	2	4.8	1	1	II	0
m	HCV	Enriched	X	X	X	X		8	1	1	3	5.8	1	1	IIIB	0
m	HBV	Depleted		X	X		X	1	1	1	1	6.7	2	0	IIIA	0
m	HBV	Enriched	X	X	X	X		23	0	1	3	8	1	0	IB	0
m	HBV	Compartmentalized	X	X	X		X	37	0	1	3	1.3	1	1	II	0
m	HBV	Depleted	X	X	X		X	8	1	0	2	16	1	1	II	0
f	HBV	Compartmentalized	X	X	X		X	26	0	0	3	10.5	1	1	II	0
m	SLD	Depleted	X	X	X		X	21	0	1	2	2.5	1	1	II	0
m	SLD	Depleted	X	X	X	X		3	0	0	3	2	1	0	IA	0
f	HCV	Enriched	X	X	X	X		12	0	1	2	1.5	1	0	IA	0
f	HCV	Enriched	X	X	X	X		5	0	1	2	4.5	1	0	IB	0
