# Supplementary material for: Spatial single-cell profiling and neighbourhood analysis reveal the determinants of immune architecture connected to checkpoint inhibitor therapy outcome in hepatocellular carcinoma
Source: Gut. 2024 Sep 30;74(3):e332837. doi: 10.1136/gutjnl-2024-332837 (PMC11874287; doi:10.1136/gutjnl-2024-332837)
Supplement: online supplemental table 3 [file gutjnl-74-3-s004.pdf]

Supplementary table 3: ICI cohort

Patient	Biopsy or resectate	No. Of ROIs	Area covered by tissue [mm²]	Sex	Age [years]	Etiology	Immunotype	BCLC stage	Child Pugh score	ECOG at baseline	Therapy regimen	Drug(s)	Line	Best Response (RECIST v1.1)	Response=1	PFS [months]	OS [months]	Censored [0=yes]	Cirrhosis [1=yes]	AFP [ng/dl]
ICI_01	B	1	1.676402	f	81	HCV	Enriched	2	5	0	ICB_combo	Nivolumab + ipilimumab	2	PR	1	1.809210526	9.733333333	1	1	41891
ICI_02	B	3	1.676402	f	78	HCV	Depleted	3	5	1	ICB_TKI_combo	Nivolumab + ipilimumab + cabozantinib	2	SD	0	4.078947368	21.3	1	1	5.4
ICI_03	B	1	0.901016	m	59	ALD	Depleted	3	5	0	ICB_TKI_combo	Nivolumab + cabozantinib	2	SD	0	5.493421053	8.366666667	1	1	2218
ICI_04	B	1	1.4802	m	57	NA	Compartmentalized	3	5	0	ICB_mono	Tislelizumab	3	SD	0	2.664473684	12.06666667	1	0	38
ICI_05	B	2	1.914995	m	61	ALD	Enriched	2	12	0	ICB_mono	Nivolumab	1	PD	0	1.233333333	1.533333333	1	1	385
ICI_06	B	1	1.208461	m	79	ALD	Compartmentalized	3	5	1	ICB_TKI_combo	Capmatinib + spartalizumab	2	SD	0	2.730263158	16.66666667	0	1	106
ICI_07	B	1	1.441754	m	57	HCV	Compartmentalized	4	11	2	ICB_mono	Nivolumab	2	PD	0	2.2	2.9	1	1	64.7
ICI_08	B	2	1.908018	m	71	HCV	NA	2	6	0	ICB_mono	Pembrolizumab	2	PD	0	1.4	26.6	0	1	5.4
ICI_09	B	2	1.895166	m	56	NA	Depleted	3	8	1	ICB_mono	Nivolumab	2	SD	0	1.133333333	1.733333333	1	0	50000
ICI_10	B	2	1.871471	m	66	HCV	Enriched	3	5	1	ICB_mono	Nivolumab	2	PR	1	39.36666667	39.36666667	0	1	5630
ICI_11	B	1	1.473947	m	57	HCV	Compartmentalized	3	7	1	ICB_mono	Nivolumab	2	PD	0	0.86	0.866666667	1	1	2279
ICI_12	B	2	1.620788	m	68	SLD	Depleted	4	10	2	ICB_mono	Nivolumab	2	PR	1	3.07	4.266666667	1	1	24.8
ICI_13	B	2	1.904014	m	65	SLD	Compartmentalized	3	7	1	ICB_mono	Nivolumab	1	PD	0	4.266666667	4.866666667	1	1	66
ICI_14	B	2	1.438137	m	73	ALD	Compartmentalized	2	5	0	ICB_mono	Pembrolizumab	1	SD	0	9.01	34.77	0	1	2
ICI_15	B	3	1.029179	m	69	HBV	Depleted	2	6	0	ICB_combo	Tremelimumab + durvalumab	1	SD	0	5.526315789	10.46666667	1	1	4.1
ICI_16	B	2	1.799427	m	74	NA	NA	2	6	0	ICB_TKI_combo	Nivolumab + cabozantinib	2	SD	0	8.289473684	14.96666667	1	0	6984
ICI_17	B	2	1.683957	m	79	ALD	Compartmentalized	3	5	1	ICB_TKI_combo	Capmatinib + spartalizumab	2	SD	0	2.730263158	16.66666667	0	1	106
ICI_18	B	2	2.188534	f	71	HCV	Enriched	3	5	1	ICB_TKI_combo	Capmatinib + spartalizumab	2	SD	0	31.84210526	34.53333333	0	0	15616
ICI_19	B	2	2.153869	f	71	HCV	Enriched	3	5	1	ICB_TKI_combo	Capmatinib + spartalizumab	2	SD	0	31.84210526	34.53333333	0	0	15616
ICI_20	B	1	2.172107	f	63	NA	Compartmentalized	3	5	1	ICB_mono	Spartalizumab	2	SD	0	3.684210526	18.46666667	0	0	3.5
ICI_21	B	2	2.003066	m	71	ALD	Compartmentalized	3	6	0	ICB_TKI_combo	Nivolumab + cabozantinib	2	PR	1	25	35.4	1	1	14949
ICI_22	B	1	1.911489	f	63	NA	Depleted	3	5	1	ICB_mono	Spartalizumab	2	SD	0	3.684210526	18.46666667	0	0	3.5
ICI_23	B	1	2.098004	m	74	NA	Enriched	2	6	0	ICB_TKI_combo	Nivolumab + cabozantinib	2	SD	0	8.289473684	14.96666667	1	0	6984
ICI_24	B	2	1.596961	m	79	SLD	Depleted	3	5	1	ICB_mono	Spartalizumab	2	SD	0	3.618421053	8.8	0	0	2.6
ICI_25	B	2	1.95248	m	64	HCV	Enriched	3	5	0	ICB_TKI_combo	Nivolumab + cabozantinib	2	PR	1	13.65131579	23.16666667	1	0	92.4
ICI_26	B	1	1.406807	m	71	HCV	Compartmentalized	3	6	1	ICB_TKI_combo	Spartalizumab + sorafenib	1	SD	0	6.644736842	9.633333333	1	0	NA
ICI_27	B	3	1.320579	m	75	ALD	Depleted	2	5	0	ICB_TKI_combo	Nivolumab + cabozantinib	2	SD	0	5.493421053	9.5	1	1	3.2
ICI_28	B	2	1.464742	m	78	ALD	Depleted	3	5	0	ICB_mono	Spartalizumab	2	SD	0	3.453947368	8.266666667	1	0	NA
ICI_29	B	2	1.673855	f	71	HBV	Depleted	3	5	0	ICB_TKI_combo	Nivolumab + avadomide	2	SD	0	3.914473684	8.566666667	1	1	16
ICI_30	B	4	1.778687	m	59	ALD	Depleted	3	5	0	ICB_TKI_combo	Nivolumab + cabozantinib	2	SD	0	5.493421053	8.366666667	1	1	2218
ICI_31	B	1	1.568587	f	79	HCV	Compartmentalized	2	5	0	ICB_TKI_combo	Spartalizumab + sorafenib	1	SD	0	2.828947368	18.93333333	0	1	NA
ICI_33	B	2	1.940899	m	65	HCV	Compartmentalized	3	6	0	ICB_mono	Durvalumab	1	PD	0	1.677631579	5.666666667	1	1	16.9
ICI_35	B	1	1.25594	m	74	HCV	Enriched	3	5	1	ICB_TKI_combo	Capmatinib + spartalizumab	2	SD	0	7.894736842	10.76666667	1	1	50000
ICI_37	B	3	2.046403	m	70	ALD	Enriched	3	5	0	ICB_combo	Tremelimumab + durvalumab	1	SD	0	3.486842105	8.866666667	1	0	69.4
ICI_38	B	2	1.009866	m	78	ALD	Depleted	3	5	0	ICB_mono	Spartalizumab	2	SD	0	3.453947368	8.266666667	1	0	NA
ICI_39	B	1	1.507454	m	73	ALD	Enriched	3	6	0	ICB_mono	Spartalizumab	2	SD	0	2.664473684	3.366666667	1	0	143
ICI_32	R	3	3.422029	m	74	SLD	Compartmentalized	3	6	1	ICB_mono	Nivolumab	2	PD	0	1.9	22.16666667	0	1	132.4
ICI_34	R	3	2.978783	f	72	NA	Depleted	3	6	0	ICB_mono	Nivolumab	2	PD	0	2.233333333	2.9	1	0	51.3
ICI_36	R	3	2.939962	m	73	HCV	Depleted	3	8	1	ICB_mono	Nivolumab	2	PD	0	2.833333333	3.433333333	1	1	183.3
ICI_40	R	3	4.297503	f	79	HCV	Depleted	2	5	0	ICB_TKI_combo	Spartalizumab + sorafenib	1	SD	0	2.828947368	18.93333333	0	1	NA
ICI_41	R	4	5.732468	m	65	HBV	Depleted	3	5	0	ICB_TKI_combo	Nivolumab + cabozantinib	2	SD	0	2.697368421	15.46666667	1	0	1.7
ICI_42	R	3	4.30366	m	77	NA	Depleted	2	5	0	ICB_combo	Tremelimumab + durvalumab	1	PD	0	1.776315789	3.1	1	0	1.9
