# Supplementary material for: Spatial single-cell profiling and neighbourhood analysis reveal the determinants of immune architecture connected to checkpoint inhibitor therapy outcome in hepatocellular carcinoma
Source: Gut. 2024 Sep 30;74(3):e332837. doi: 10.1136/gutjnl-2024-332837 (PMC11874287; doi:10.1136/gutjnl-2024-332837)
Supplement: online supplemental table 4 [file gutjnl-74-3-s005.pdf]

Supplementary table 4: Comparison of clinical cohorts

		Discovery cohort	ICI cohort	p-value	Statistical test
**Age average [years]**		62.9	70	p<0.0001	Mann-Whitney
**Sex [n(%)]**				p=0.8666	Chi-Square
	Male	42 (78)	32 (76)		
	Female	12 (22)	10 (24)		
**Liver disease [n(%)]**				p=0.000001921	Chi-Square
	HBV	12 (22)	3 (7)		
	HCV	16 (30)	15 (36)		
	ALD	14 (26)	12 (29)		
	SLD	10 (18)	4 (9)		
	HBV+HCV	2 (4)	0		
	Unknown	0	8 (19)		
**Spatial immune types [n(%)]**				p=0.04385	Chi-Square
	Depleted	31 (58.5)	17 (42.5)		
	Compartmentalized	10 (18.9)	13 (32.5)		
	Enriched	12 (22.6)	10 (25)		
