# Supplementary material for: Spatial single-cell profiling and neighbourhood analysis reveal the determinants of immune architecture connected to checkpoint inhibitor therapy outcome in hepatocellular carcinoma
Source: Gut. 2024 Sep 30;74(3):e332837. doi: 10.1136/gutjnl-2024-332837 (PMC11874287; doi:10.1136/gutjnl-2024-332837)
Supplement: online supplemental figure 1 [file gutjnl-74-3-s006.pdf]

Supplementary Figure 1

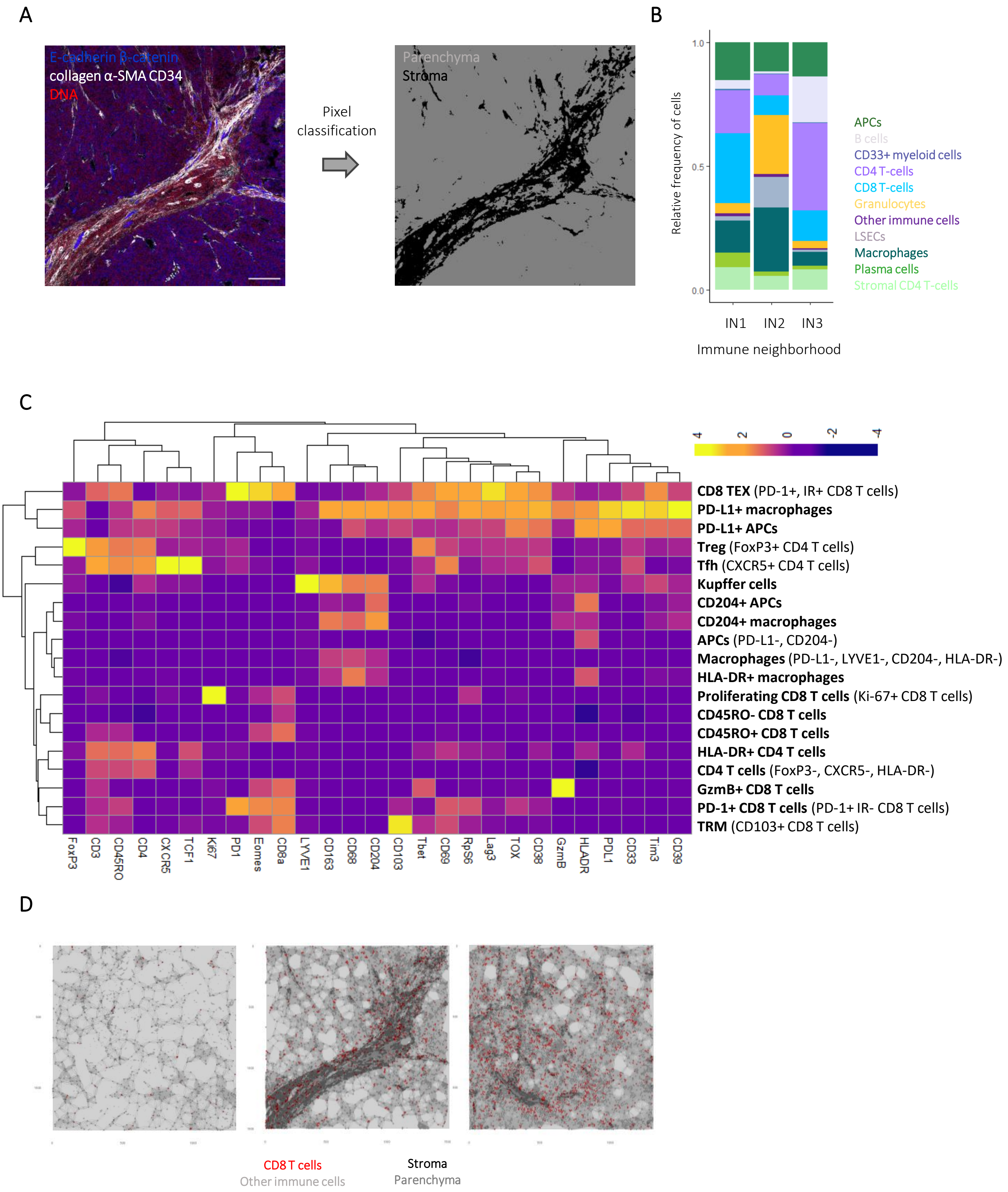

Supplementary Figure 1 (related to Figures 1, 2 and 3):
A: Example composite image (left) visualizing epithelial markers (blue) and stromal markers (white) used to generate stroma masks (right). Scale bar indicates 200 µm. **B:** Stacked bar plot showing the distribution of immune cell types in each immune neighborhood. **C:** Heatmap visualizing z-scored median marker expression of gated immune subsets in the discovery cohort. **D:** Example images showing the spatial distribution of CD8 T cells (red) among other immune cells (gray) between the tumor stroma (dark gray) and parenchyma (light gray).

Supplementary Figure 2

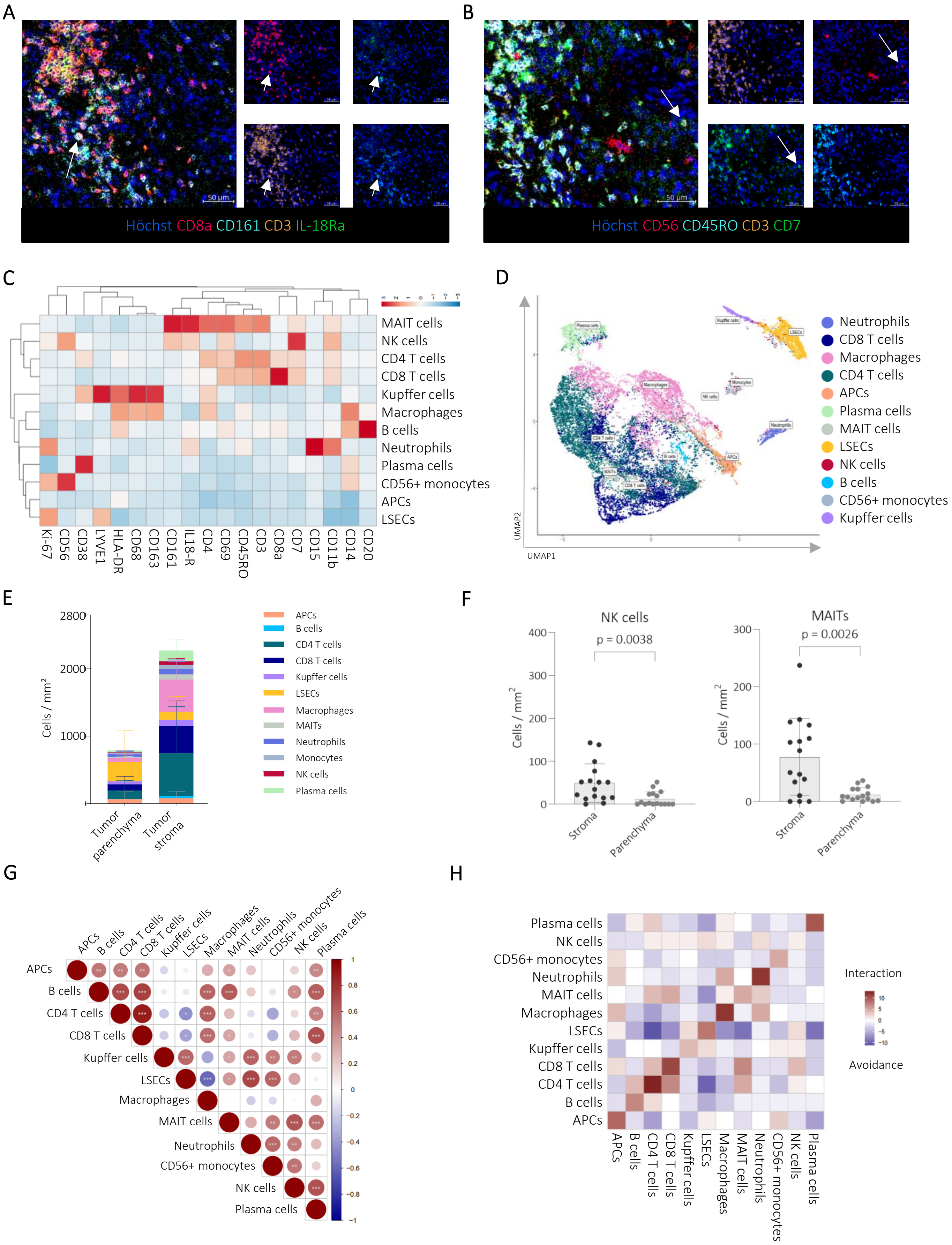

Supplementary Figure 2 (related to Figure 2): NK cells and MAIT cells in the HCC microenvironment.
A and B: Example composite image visualizing MAIT cells (A) and NK cells (B). Scale bars indicate 50µm. C: Heatmap showing normalized and z-scored expression of indicated marker proteins per immune cell type. D: UMAP colored by immune cell type visualizing their behavior in low dimensional space. Each dot represents a cell. E: Stacked bar plot visualizing mean immune cell type densities in the tumor parenchyma compared to intratumor stroma. F: Boxplots comparing NK cells (left) and MAIT cell (right) densities between tumor parenchyma and stroma. Each dot represents an ROI. Wilcoxon tests were used to assess statistical significance. G: Heatmap of Spearman's correlation between cell types. H: Heatmap visualizing immune cell interactions. Color coding represents the number of ROIs with significant interactions (red) or avoidances (blue) of immune cell pairs.
*p<0.05, **p<0.01, ***p<0.001.

Supplementary Figure 3

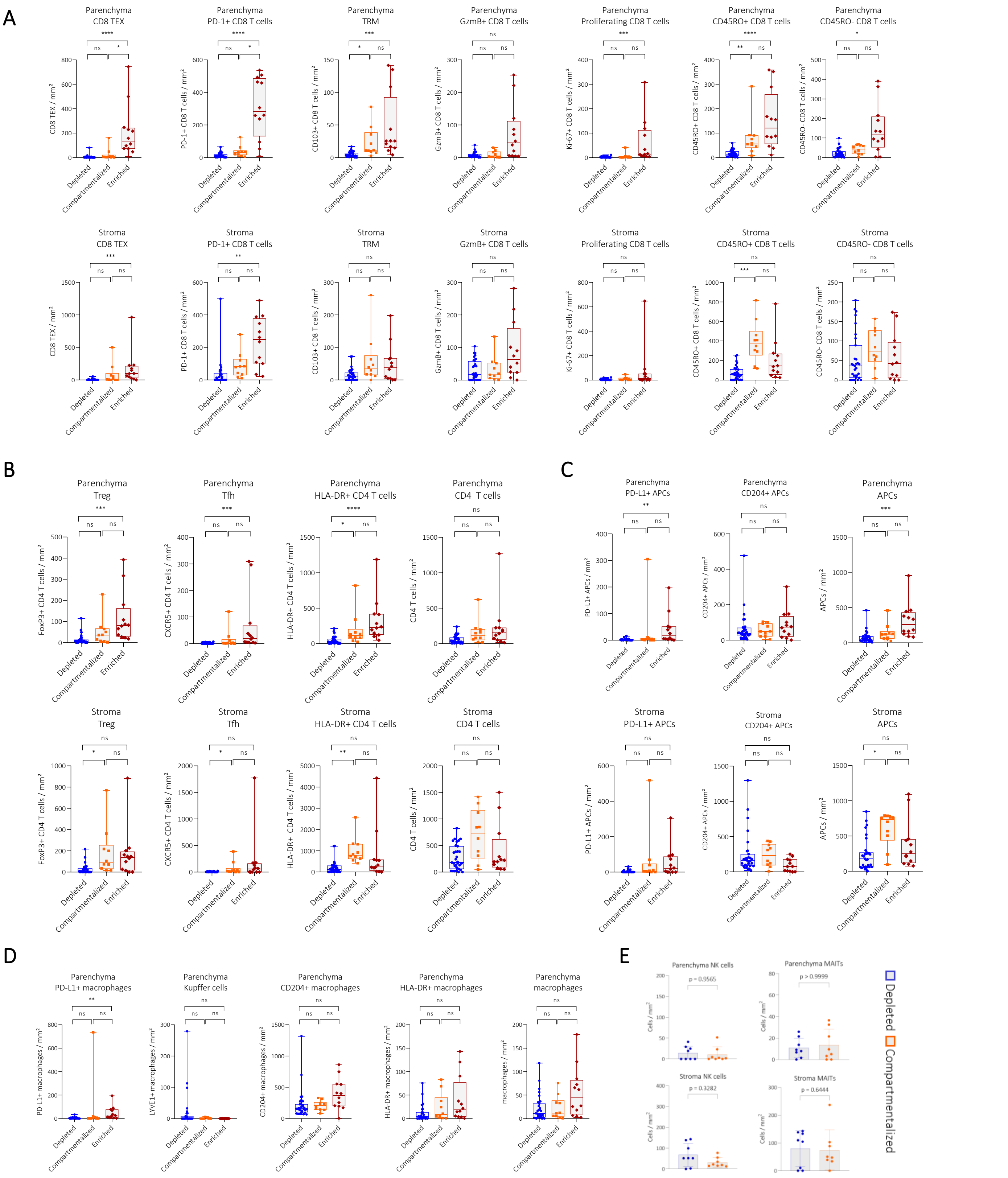

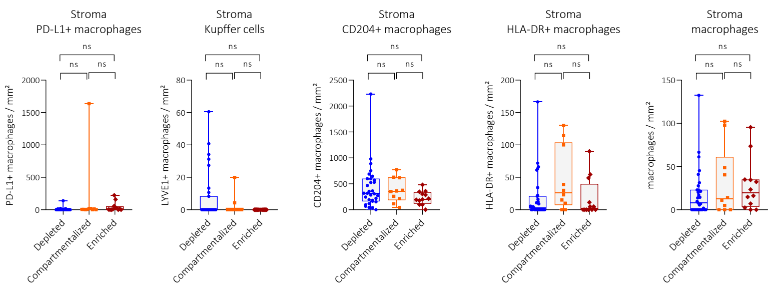

Supplementary Figure 3 (related to Figure 3):
Boxplots comparing density of CD8 T-cell subsets (A), CD4 T-cell subsets (B), APC subsets (C), macrophage subsets (D) and NK and MAIT cells (E) in the parenchyma (top row) and stroma (bottom row) compartments of the tumor ROIs between spatial immune types. Mann-Whitney tests were used to assess significance of pairwise comparisons and p-values were Bonferroni corrected for multiple comparisons.
ns = not significant, *p<0.05, **p<0.01, ***p<0.001, ****p<0.0001.

Supplementary Figure 4

A

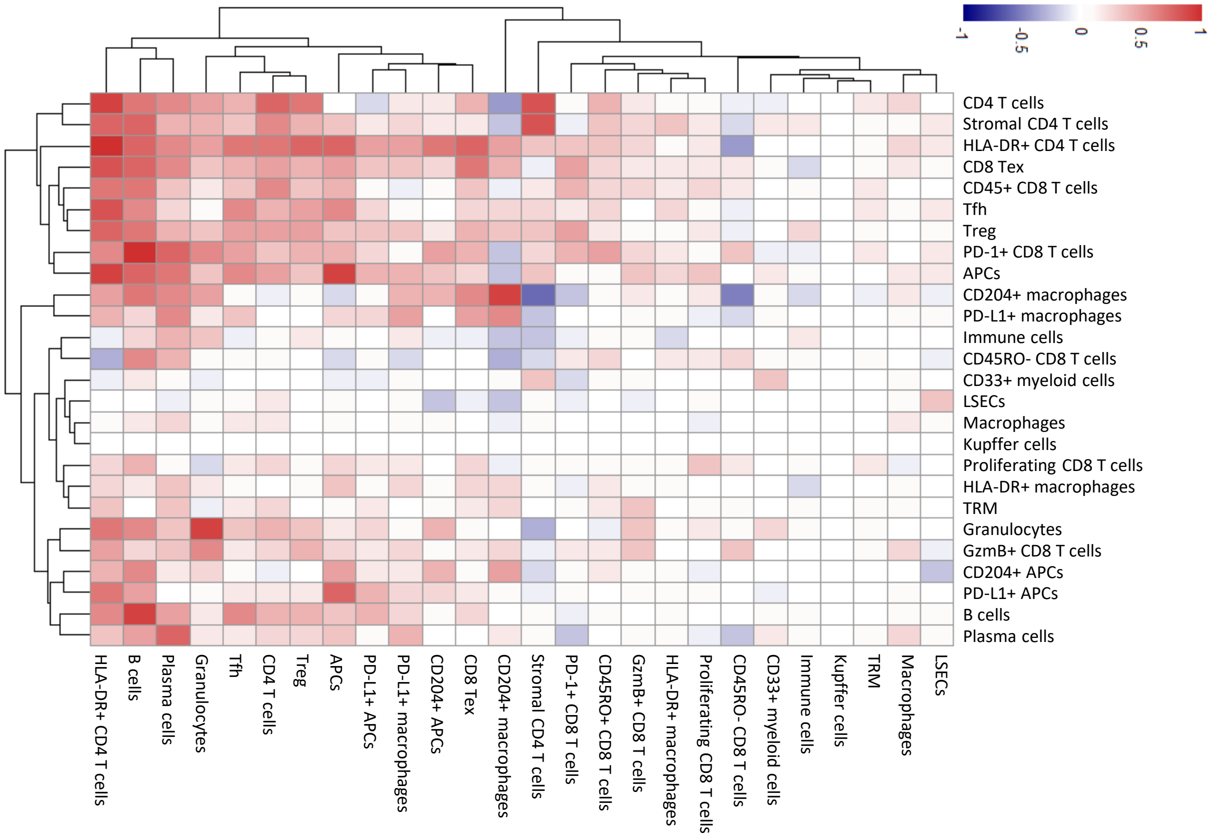

B

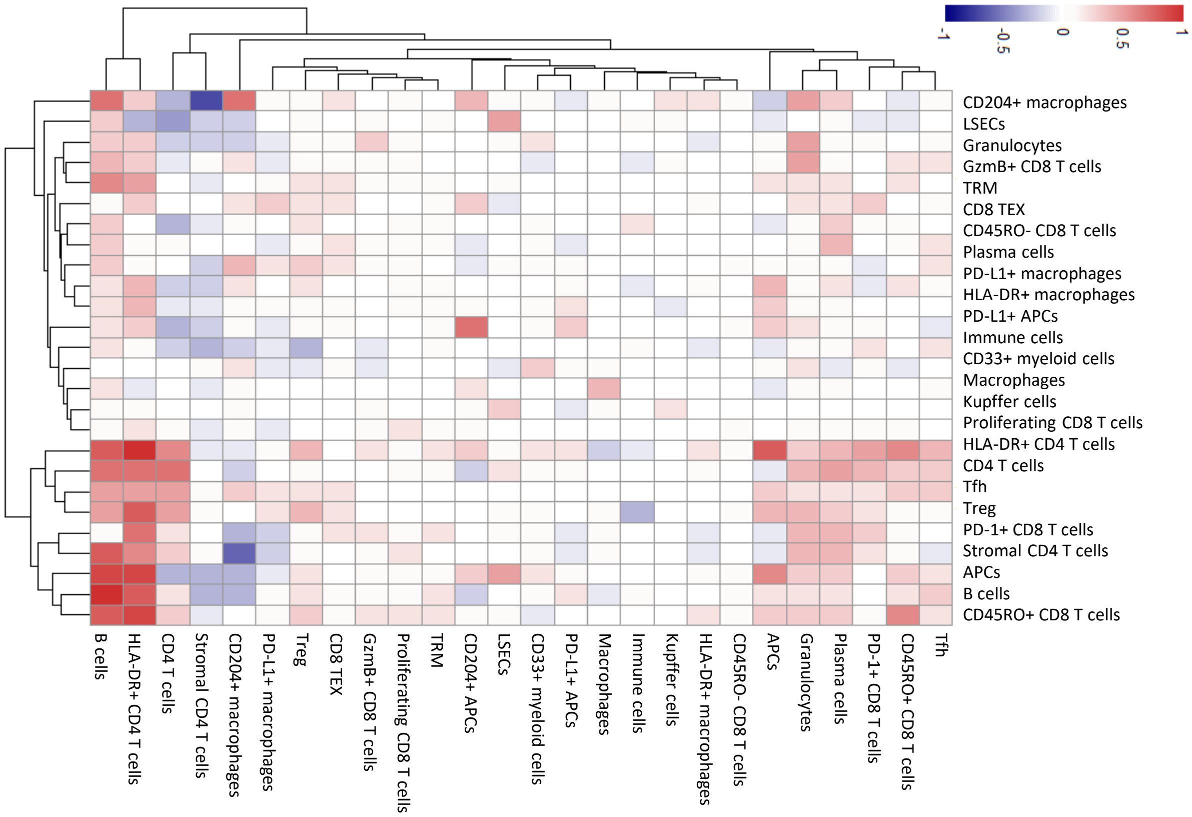

Supplementary figure 4 (related to Figure 3):
Heatmap visualizing immune cell interactions of enriched (A) and compartmentalized (B) patients. Color coding represents the fraction of patients with significant interactions (red) or avoidances (blue) of immune cell pairs within the tumor ROIs.

Supplementary Figure 5

A

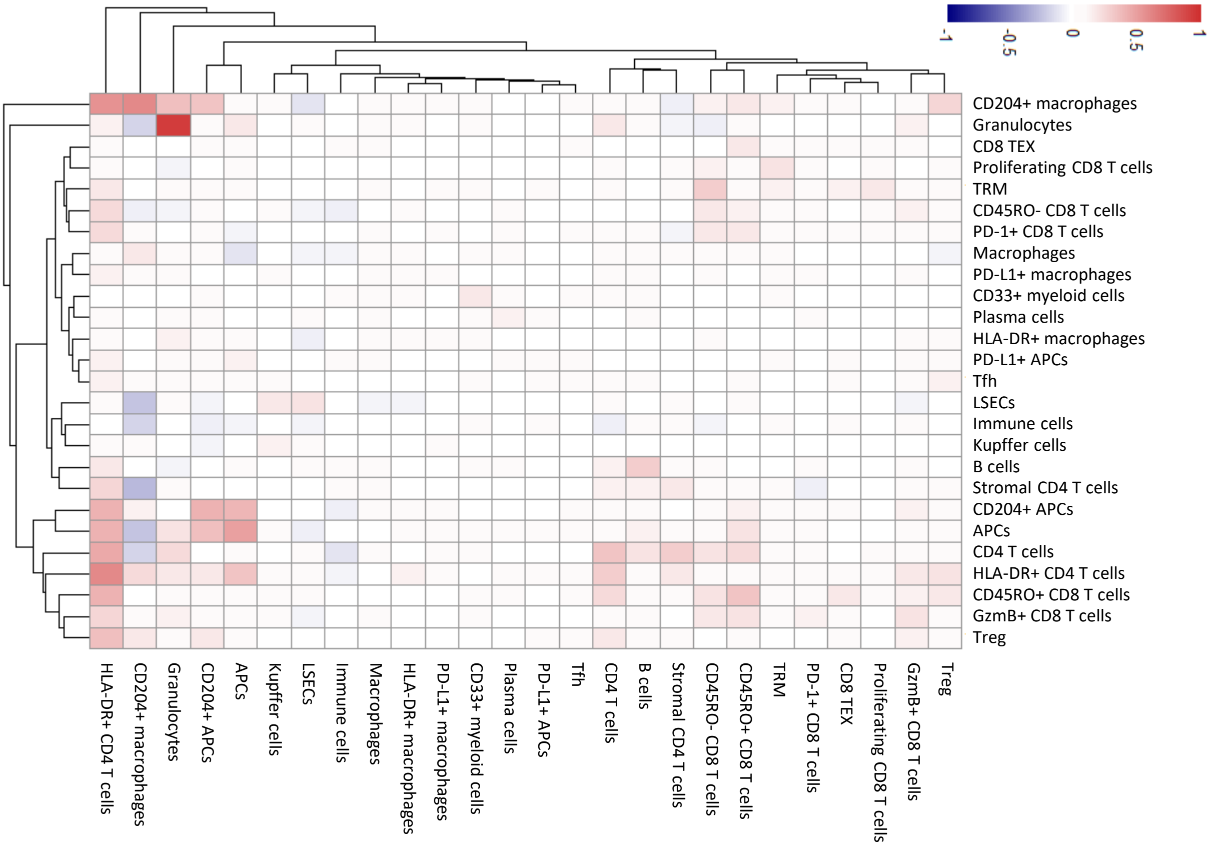

B

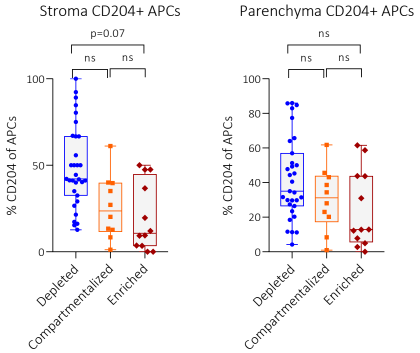

Supplementary Figure 5 (related to Figure 3):
A: Immune cell interactions of depleted patients. Color coding represents the fraction of patients with significant interactions (red) or avoidances (blue) of immune cell pairs within the tumor of depleted patients. **B:** Boxplots showing frequency of CD204+ APCs in the stroma (left) and parenchyma (right) of all APCs. Each dot represents a patient. Mann-Whitney tests were used to assess for pairwise statistical significance and p-values were Bonferroni corrected to account for multiple comparisons.
ns = not significant, *p<0.05, **p<0.01, ***p<0.001, ****p<0.0001.

Supplementary Figure 6

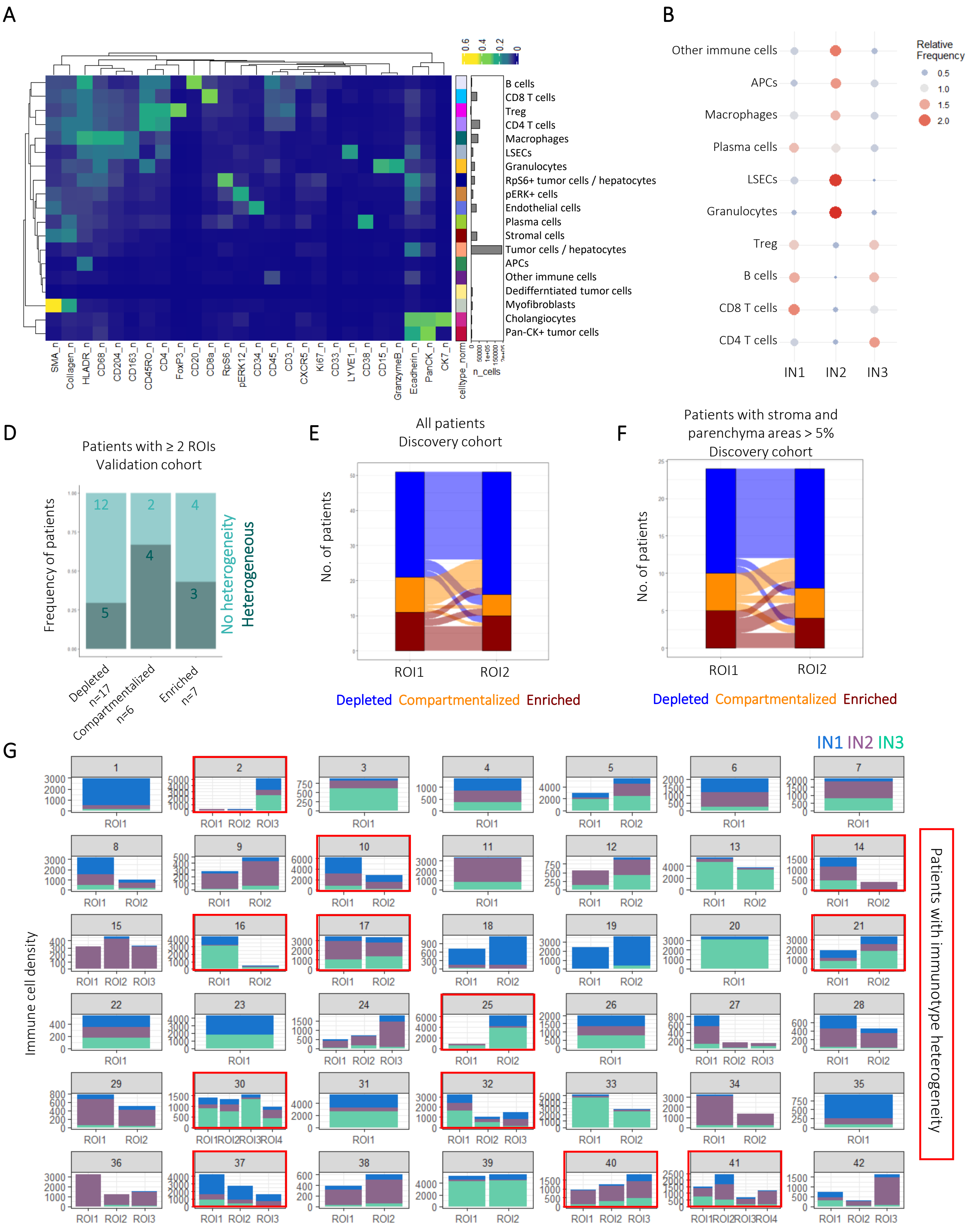

Supplementary Figure 6 (related to Figure 6):

A: Heatmap visualizing mean marker expression of identified cell types in the HCC tumor microenvironment in the ICI cohort. B: Bubble heatmap showing relative frequencies of immune cell types within the identified immune neighborhoods in the ICI cohort. C: Stacked bar plots showing the frequency of patients with and without immunotype hetrogeneity between immunotypes in the ICI cohort. D and E: Sankey plot visualizing the immunotype heterogeneity between the initial (ROI1) and remeasured (ROI2) ROIs in the discovery cohort (D) and in the discovery cohort after exclusion of ROIs with less than 5% stroma or parenchyma. F: Stacked bar plots visualizing immune neighborhood contributions of each ROI of each patient in the therapy cohort. Patients with immunotype heterogeneity are encircled in red.

Supplementary Figure 7

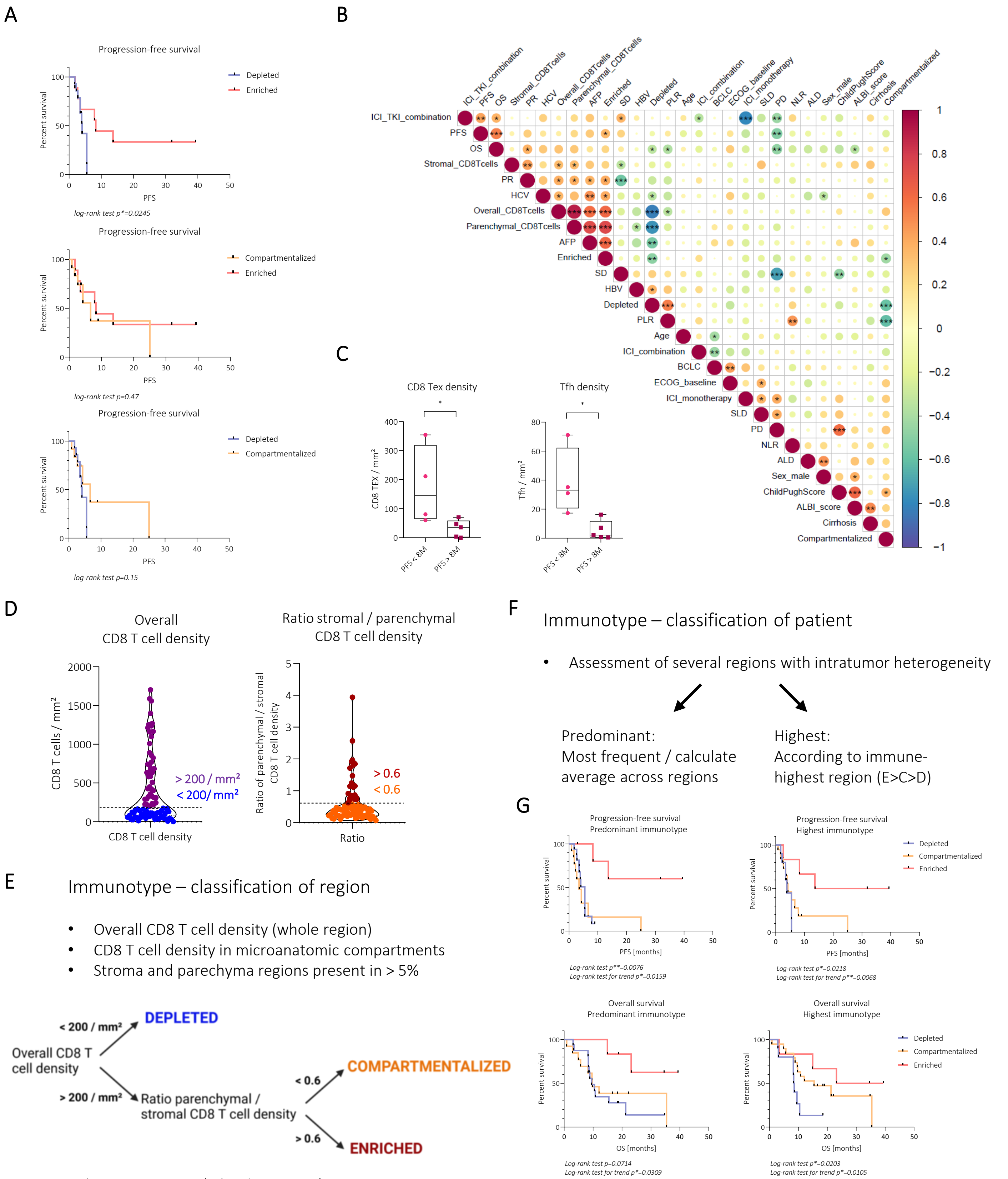

Supplementary Figure 7 (related to Figure 7):

A: Pairwise Kaplan-Meier survival analysis of spatial immune types. Log-rank tests were used to assess statistical significance. **B:** Heatmap visualizing pairwise correlations between shown parameters. Correlation between two continuous variables was assessed by Spearman correlation. Correlation between two binary variables or one binary and one continuous variable was assessed by Wilcoxon tests. Benjamini-Hochberg correction was used to adjust p-values for multiple comparisons. PLR = platelet-to-lymphocyte ratio, NLR = neutrophil-to-lymphocyte ratio. **C:** Boxplots comparing CD8 TEX and Tfh densities between enriched patients with PFS longer and shorter than 8 months. Mann-Whitney tests were used to assess statistical significance. D: Flowchart depicting prerequisites abd optimized parameters for immunotype classification of single intratumora regions E: Violin plots visualizing the overall CD8 T cell density (left) and ratio between stromal and parenchymal CD8 T cell density from all HCC patients incuded in the study. Each dot represents a patient and is colored accrding to the chosen cut-off value which is depicted by the dotted line. F: Box specifying the options for immunotype classification of patients if ITH was detected. It can be achieved by choosing the predminant (A) or the highest immuntype (B). G: Kaplan-Meier survival curved depicting profression-free survival (top) and overall survival (bottom) of patients classified with the optimized parameters and assigned the predominant (left) or highest (right) immunotype if ITH was detected.
